# Bioactive Compounds and In Vitro Health-Promoting Activity of the Fruit Skin and Flesh of Different Haskap Berry (*Lonicera caerulea* var. *kamtschatica* Sevast.) Cultivars

**DOI:** 10.3390/ijms26146618

**Published:** 2025-07-10

**Authors:** Natalia Żurek, Stanisław Pluta, Michał Świeca, Leszek Potocki, Łukasz Seliga, Ireneusz Kapusta

**Affiliations:** 1 Department of Food Technology and Human Nutrition, Faculty of Technology and Life Sciences, University of Rzeszow, 4 Zelwerowicza St., 35-601 Rzeszow, Poland; ikapusta@ur.edu.pl; 2 Department of Horticultural Crop Breeding, National Institute of Horticultural Research (InHort), Konstytucji 3 Maja 1/3 St., 96-100 Skierniewice, Poland; stanislaw.pluta@inhort.pl (S.P.); lukasz.seliga@inhort.pl (Ł.S.); 3 Department of Food Chemistry and Biochemistry, University of Life Sciences in Lublin, 8 Skromna St., 20-704 Lublin, Poland; michal.swieca@up.lublin.pl; 4 Department of Biotechnology, University of Rzeszow, 1 Pigonia St., 35-310 Rzeszow, Poland; lpotocki@ur.edu.pl

**Keywords:** haskap berry, health-promoting activity, polyphenols compounds, iridoids, UPLC, physicochemical properties

## Abstract

The study focused on the distribution of polyphenolic compounds, iridoids, organic acids, and sugars, as well as in vitro antioxidant, anti-inflammatory, antidiabetic, antiproliferation, and antibacterial potential, and physicochemical properties between the skin and flesh of 10 haskap berry (*Lonicera caerulea* var. *kamtschatica* Sevast.) cultivars. It was found that the content of individual bioactive compounds significantly depended on the fruit cultivar and the analyzed morphological part. Anthocyanins, kaempferol derivatives and iridoids dominated in the skin, which significantly correlated with most of the analyzed health-promoting properties. In turn, the flesh showed a higher content of quercetin derivatives, sugars and organic acids. No differences were found in the content of phenolic acids, flavan-3-ols and antibacterial activity. The most beneficial properties were shown for the cultivar (cv.) ‘Honeybee’. The study suggests that haskap fruit skin is a valuable raw material for use in the pharmaceutical and food industries.

## 1. Introduction

Previously named blue honeysuckle, now haskap berry or honeyberry (*Lonicera caerulea* L.) is a shrub belonging to the Caprifoliaceae family and one of 180 shrubs belonging to the honeysuckle genus *Lonicera* L. As a medicinal and useful plant, it is widely cultivated in Russia, China, Japan and, to a lesser extent, in North America and Europe. Haskap berry bushes can reach heights of 1 to 3 m [1]. The fruits are fleshy and ripen depending on the region of cultivation between May and July, reaching up to 2 cm in length and 1 cm in width and weighing from 0.3 to 2.0 g. After about 3 years of cultivation, a yield of about 0.5 kg of fruit is achieved from each bush. The berries have a sweet and sour taste, although sometimes with a bitter aftertaste [2].

The health-promoting properties of haskap berries have recently been widely analyzed. Some studies have shown in vivo cardioprotective and neuroprotective effects of fruit extracts [3,4]. In addition, active fractions have been studied for antiproliferation activity against prostate and liver cancer cells [5,6], their potential in the treatment of diabetes [7,8], and their ability to alleviate oxidative stress and inflammation in the intestines and regulate the growth of intestinal microbiota in mice [9]. The above-mentioned health-promoting effects of haskap berries are mainly attributed to the polyphenolic compounds present in them. Their composition is dominated by anthocyanins (50–95% of all polyphenolic compounds), among which cyanidin 3-*O*-glucoside is present in the highest concentration. The remaining groups of phenolic compounds include phenolic acids (caffeic, chlorogenic, *p*-coumaric acids), flavonols (quercetin, kaempferol) and flavan-3-ols (epicatechin, procyanidins) [2,10,11]. In addition to polyphenolic compounds, it has been proven that iridoids, such as loganic acid, loganin, severoside, secoxyloganin and secologanin, that are present in haskap berries can influence their toxicological effects [9,12].

However, it is known that bioactive compounds are not evenly distributed in the individual morphological parts of the fruit. Despite this, the accumulation of polyphenolic compounds in the skin and flesh of only one cultivar of haskap berry has been assessed so far, but these results were from pomace after pressing [13,14]. It was shown that the main anthocyanins and flavonols are present in the skin pomace, but no differences were found in the distribution of iridoids. Therefore, the limited knowledge on the distribution of bioactive components in haskap berries and its effect on health-promoting properties emphasizes the need for further research.

For this reason, the aim of the study was to quantitatively and qualitatively analyze the content of polyphenolic compounds (anthocyanins, phenolic acids, flavonols, flavanols) and iridoids, and to assess the health-promoting potential in the form of antioxidant, anti-inflammatory, antidiabetic, antiproliferation and antibacterial activity of the skin and flesh of 10 cultivars of haskap berries grown in Poland. In addition, we analyzed basic chemical parameters, such as the content of sugars and organic acids, as well as physicochemical parameters. We believe that such a research profile will allow us to indicate the morphological fruit parts and cultivars of haskap berries with the highest biological potential, which will be of great importance in the future design of nutraceuticals, functional foods, cosmetics or dietary supplements.

## 2. Results and Discussion

### 2.1. Physicochemical Properties

The results for physicochemical properties are presented in Table 1. The average value of titratable acidity (TA) for the skin of haskap berries was 1.2 times higher than for the flesh. The lowest TA value was found for the flesh of the cv. ‘Boreal Beast’ (2.06 g citric acid/100 g), and the highest for the ‘Vostorg’ skin (3.18 g citric acid/100 g). Higher values for pomace from both the flesh and skin of haskap berries were shown by Oszmiański et al. [13] (3.5 and 3.0 g citric acid/100 g, respectively). The value of this parameter is largely determined by the fruit ripening (harvest date), which also determines the taste of the fruit [1]. A parameter that has an equally high effect on the quality characteristics of the fruit is soluble solids content (SSC). The lowest value was found for the flesh of the cv. ‘Boreal Blizzard’ (10.22 °Brix), and the highest for the skin of the cv. ‘Usłada’ (16.04 °Brix). The SSC value may depend on the time and conditions of fruit storage. Dziedzic et al. [15] showed that 14-day storage of haskap berries in refrigerated conditions (+4 °C) causes a 1.2-fold decrease in the SSC value. For the last two parameters, dry matter (DM) and ash, higher average values were recorded for the skin of the fruit, 15.12 g/100 g and 0.58%, respectively. The mineral composition of the fruit may depend on the cultivar and growth conditions, but also on the morphological fruit part [1].

### 2.2. Content of Iridoid and Polyphenolic Compounds

In the haskap berry fruits, a total of 34 compounds from two groups were identified: polyphenols and monoterpenoids (iridoids), as presented in Table 2, Table 3 and Table S1, Figures S2–S7. The detected compounds can be classified into five subgroups, i.e., anthocyanins (10 compounds), flavanols (14), phenolic acids (three compounds), flavan-3-ols (one) and iridoids (six compounds). Quantitatively, the content of polyphenolic compounds in the haskap berry skin ranged from 68.61 to 118.01 mg/g, while in the flesh, from 26.31 to 37.02 mg/g. For both the skin and the flesh, the highest value was found for the cv. ‘Honeybee’ and the lowest for ‘Vostrog’. In the quantitative profile of polyphenolic compounds in the skin, 85.5% of the average composition was anthocyanins, 11.1% flavonols, 3.1% phenolic acids and 0.2% flavan-3-ols. In turn, the percentage composition of the flesh was as follows: 72.3% > 19.0% > 8.5% > 0.2%. The types of polyphenolic compounds were similar to those previously reported [6,8,12]. In the quantitative profile of pomace from whole fruits of the cv. ‘Vostorg’, Oszmiański et al. [13] showed 24.4 mg/g of polyphenolic compounds, including 2.0-fold higher and 1.2-fold lower content in pomace from the skin and flesh, respectively. Apart from the morphological parts of the fruit, it was previously shown that the polyphenolic composition of haskap berries may depend on the date of fruit harvest [16], cultivation conditions and the degree of exposure to abiotic factors [17].

The dominant group of compounds included anthocyanins, which were located mainly in the skin of haskap berries. Their average content was 73.46 mg/g, 3.3 times higher than in the flesh and significantly dependent (*p* < 0.05) on the analyzed cultivar. The highest value was recorded for the skin of the cv. ‘Honeybee’ (98.50 mg/g). Previously, 14.7, 38.4 and 9.9 times higher content of anthocyanins in the fruit skin than in the flesh were confirmed for grapes [18], highbush blueberries [19] and Saskatoon berries [20], respectively. In our previous studies of whole haskap berries, the anthocyanin content ranged from 2.0 to 3.2 mg/g [11]. A significantly higher concentration of anthocyanins in whole haskap berries was found by Guo et al. [21] (59.1 mg/g), showing 4.6 and 12.7-fold higher content of these compounds compared to blackberry and cranberry, respectively. It has been previously shown that a key marker of the anthocyanin profile of haskap berries is the content of cyanidin 3-*O*-glucoside, which accounted for 71.1–96.0% of total anthocyanins [6,8,21]. This is also confirmed by our research, where this compound constituted, on average, 83.8 and 82.8% of the anthocyanin composition of the skin and flesh, respectively.

The next group consisted of flavonols. The skins of the haskap berry fruits contained from 5.70 (‘Aurora’) to 14.11 (‘Honeybee’) mg/g of flavonols, while the flesh contained from 4.92 (‘Jugana’) to 7.50 (‘Honeybee’) mg/g. The content of flavonoids in the skin and flesh was higher than the content previously reported for the whole fruits of the cvs. ‘Aurora’ (0.5 mg/g) and ‘Loni’ (1.57 mg/g) analyzed by Senica et al. [17] and Craciunescu et al. [6], respectively. Similarly to anthocyanins, flavonols were not evenly distributed in the haskap berry fruits. Kaempferol derivatives were identified mainly in the skins of the studied cultivars, while quercetin derivatives dominated in the flesh. The highest concentration of kaempferol 3-*O*-rhamnoside was found in the skin (on average, 65.1% of total flavonoids), while in the flesh, quercetin 3-*O*-rhamnoside (on average, 69.2% of total flavonoids). Previously, Senica et al. [17] showed that the content of flavonols in haskap berries depends on the degree of their exposure to the sun.

In the phenolic acids, comparable content was found for both the skin and the flesh of the haskap berry. The content of phenolic acids ranged from 1.52 (‘Aurora’) to 4.78 (‘Honeybee’) mg/g in the skin, and from 1.64 (‘Aurora’) to 3.32 (‘Honeybee’) mg/g in the flesh. So far, 0.8 mg/g of phenolic acids has been detected in whole haskap berries, where chlorogenic acid constituted 68.7–89.1% of all these compounds [6,12,13,17]. In our study, both in the fruit skin and in the flesh, chlorogenic acid was also identified in the highest concentration (86.0 and 73.8% of total phenolic acids, respectively). Oszmiański et al. [13] reported results comparable to those in our study for the content of phenolic acids in pomace from whole fruits and from the skin of haskap berries, and 2.1 times lower for pomace from the flesh.

The lowest content of flavan-3-ols was identified in the skin, ranging from 0.11 (‘Aurora’) to 0.42 (‘Honeybee’) mg/g, and from 0.04 (‘Vostorg’) to 0.10 (‘Boreal Blizzard’, ‘Honeybee’, ‘Jugana’) mg/g in the flesh of the haskap berry. In our studies, we identified only one compound belonging to this group—procyanidin dimer type-B. Previously, the presence of (+)-catechin and (−)-epicatechin in the haskap berry fruit was also reported [12,22]. Higher content of flavan-3-ols for whole fruits (0.1–0.9 mg/g) was also found [14]. The differences shown in the composition and content of flavan-3-ols in the haskap berry fruits may be determined by the genotype and the fruit maturity stage, which was previously confirmed by Kucharska et al. [12].

The last group of identified compounds were iridoids, which apart from haskap berries, have also been detected in cranberries, dogwoods and blackberries [12,23,24]. Oszmiański and Kucharska [14] examined pomace from the skin and flesh and found that iridoids were evenly distributed in haskap berries, in contrast to polyphenolic compounds. In our study, a higher total content of iridoids was detected in the skin than in the flesh. The content of iridoids in the skin ranged from 8.89 to 28.31 mg/g, while in the flesh, it ranged from 7.27 to 19.94 mg/g. The highest contents were found for the ‘Jugana’ (skin) and ‘Honeybee’ (flesh), while the lowest were found for ‘Usłada’ (skin) and ‘Boreal Blizzard’ (flesh). So far, only a few studies have identified iridoids in haskap berry fruits. For freeze-dried cv. ‘Wojtek’ fruits, using pomace from the skin and from the flesh, the total iridoid content was found to be 5.3, 4.2 and 2.9 mg/g, respectively, with the dominant compounds being loganic acid and loganin [14]. In turn, Kucharska et a. [12], in fresh ‘Vostorg’ fruits, found a dominance of loganin 7-*O*-pentoside and loganic acid 7-*O*-pentoside. In our study, the main compounds identified in the skin were loganic acid and loganin (35.3% and 33.3% of total iridoid content, respectively), whereas in the fruit flesh, loganin and sweroside pentoside isomer I were dominant (44.2% and 22.5%, respectively).

### 2.3. Sugars and Organic Acids

Sugars and organic acids identified in the fruit are mainly responsible for their sensory values, and in the case of acids, also for health properties. The content of total sugars in the haskap berry flesh was 10.9 times higher than in the skin (Table 4). Two sugars were identified in the flesh of the tested cultivars–glucose, which constituted 52.1% of total sugars, and fructose. A similar trend was noted in the haskap berry skin. The dominant sugar was fructose (53.3%), while the next sugar was glucose. For the flesh, the highest sugar content was characteristic of the cv. ‘Usłada’, which may indicate that the fruits of this cultivar are relatively sweet, and for the skin, the cv. ‘Sinij Uties’ was highest. On the other hand, the lowest content was found for the cv. ‘Boreal Beast’ (flesh) and Boreal Beauty (skin). So far, Česonienė et al. [25], Molina et al. [26] and Oszmiański et al. [13], using whole fruits, reported the content of sugars as in the range 7.6–43.8 g/100 g, and the dominant sugar was also fructose, the concentration of which in fruits, according to Molina et al. [26], was up to 4.0 g/100 g. Other sugars identified in fruits included sucrose (1.2 g/100 g) [25] and sorbitol (0.1 g/100 g) [27]. So far, only one report has assessed the content of sugars in individual morphological parts of haskap berry fruits. Oszmiański et al. [13] found a 2.4-fold higher total sugar content for pomace from the skin than for pomace from the flesh, and the dominant sugar was glucose (content of 17.7 and 7.9 g/100 g, for the skin and flesh, respectively). In our study, we identified significantly higher differences between the sugar content in the skin and flesh, although in the cited paper, pomace was used for analysis, which could significantly affect the demonstrated sugar profile. Differences in the sugar content in haskap berries may also be conditioned by the date of fruit harvest and exposure to light, which was confirmed in earlier analyses [17].

In total, three organic acids were identified, with three in the skin and two in the flesh of the haskap berry fruit, including citric acid (72.7 and 59.4% of total organic acids for the skin and flesh, respectively) > quinic acid (27.3 and 22.8%) > malic acid (0 and 17.8%) (Table 4). Higher total acid content was detected for the flesh (range 11.94–48.51 g/100 g), while for the skin, this value was 0.47–1.75 g/100 g. These values significantly differed (*p* < 0.05) depending on the cultivar tested. The highest content was found for the cv. ‘Vostorg’ (both for the skin and flesh), and the lowest for the cv. ‘Boreal Beast’ (skin and flesh). So far, no studies have assessed the content of organic acids in the morphological parts of the haskap berry fruit. In whole fruits, the acids detected in the study were identified, as well as phytic, oxalic, shikimic, tartaric, and fumaric acids [17,27]. Their total amounts ranged from 1.5 to 3.9 g/100 g, and the dominant acid, as in our study, was citric acid (70.2% of the total acid content) [26]. Previously, in studies on berry fruits, it was reported that the content of organic acids was negatively correlated with light intensity, in contrast to the content of sugars. A decrease in the content of acids was also found with the increase in the content of phenolic compounds in fruits [17]. In our study, we also found a negative correlation between the total content of organic acids and phenolic compounds (r = −0.91 (skin); r = −0.87 (flesh); *p* < 0.05) (Tables S3 and S4).

### 2.4. Antioxidant Activity

All tests for antioxidant activity assessment confirmed higher antioxidant activity in the skin than in the haskap berry flesh (Table 5 and S2). The results for the fruit skin were in the range of 68.64–103.65 mmol TE/100 g (ABTS), 41.32–63.91 mmol TE/100 g (CUPRAC), 240.04–428.91 μg/mL (ChP; expressed as IC_50_), and 334.15–833.61 and 371.70–765.72 μg/mL (OH˙ and O_2_˙^−^, respectively; expressed as IC_50_). The highest antioxidant potential was found for the cv. ‘Honeybee’. Compared to the flesh of the same cultivar, the values for these tests were 2.1, 2.1, 1.8, 2.0 and 1.6 times lower, respectively.

For comparison, the antioxidant activity in the ABTS and CUPRAC whole fruit tests was in the ranges of 2.2–137.9 and 19.1–69.8 mmol TE/100 g, respectively [15,22,28]. At the same time, Oszmiański et al. [13], using pomace from the skin, showed activity of 0.65 mmol TE/100 g, including activity 1.2 times higher than for whole fruit and 1.6 times lower for pomace from the flesh. Recently, Zhang et al. [8], analyzing 20 cultivars of haskap berry, reported that it was mainly the anthocyanin fraction that determined the ability to remove ABTS radicals. The antioxidant activity in the skin and flesh of haskap berries has not been assessed so far using other tests. However, in our previous studies for whole fruits of 10 haskap berry cultivars, we showed values comparable to those obtained in this study, also proving that it was anthocyanins, mainly cyanidin 3-*O*-glucoside content, that shaped the antioxidant activity in the fruits [11]. This was also confirmed in the data published by Craciunescu et al. [6] and Guo et al. [21]. Generally, in our studies, it was noted that skin anthocyanins showed significant Pearson correlation with the ABTS test (r = 0.96; *p* < 0.05), CUPRAC (r = 0.97; *p* < 0.05), and O_2_˙^−^ (r = −0.97; *p* < 0.05) (Table S3). In the case of the flesh, the antioxidant activity was more strongly correlated with the total content of polyphenolic compounds (r = 0.91 (ABTS); r = −0.93 (OH˙); *p* < 0.05) (Table S4). However, no correlation was found between antioxidant activity and iridoid content. Earlier, Kucharska et al. [12] also noted a weak effect of iridoids on the antioxidant activity of haskap berries when measured by DPPH and FRAP methods, stating at the same time that their moderate ability to scavenge radicals probably results from their weak ability to donate hydrogen. Nevertheless, their presence in haskap berries may enhance the bioactive potential of polyphenolic compounds, exceeding the potential of other fruits containing only polyphenolic compounds.

### 2.5. Antidiabetic Activity

Several previous studies have indicated that haskap berries can serve as potential inhibitors of digestive enzymes responsible for carbohydrate hydrolysis [7,29]. The IC_50_ values of α-amylase and α-glucosidase inhibition obtained in our analyses were significantly higher for the fruit skin, where they ranged from 4.84 (Honeybee) to 11.92 (Boreal Beauty) mg/mL and from 8.35 (Honeybee) to 17.80 (Vostorg) mg/mL (Table 5 and Table S2). Compared to the flesh of the most active cultivar (Honeybee), the α-amylase and α-glucosidase inhibitory activity was 2.1 and 3.0 times lower, respectively. The results obtained were similar to previously published reports. The inhibitory activity of whole fruit extracts against α-amylase and α-glucosidase ranged from 2.4 to 50.2 mg/mL and from 39.9 to >400.0 mg/mL, respectively [16,30]. A significantly higher potential was demonstrated for the isolated polyphenolic fraction (0.3 mg/mL), showing a better result than for acarbose [7]. The authors concluded that cyanidin 3-*O*-glucoside, chlorogenic acid and catechin are responsible for such a strong effect, and the combination of the enzyme with inhibitors is stabilized by hydrophobic interactions, hydrogen bonds and ionic interactions, which results in a decrease in α-amylase activity. Importantly, this mechanism was stronger for the fruit extract than for its individual active ingredients. A different view was presented by Zhang et al. [8,31], who attributed the antidiabetic activity of haskap berries solely to the content of anthocyanins. For fractions of this group of compounds isolated from 20 cultivars of haskap berries, the values inhibiting α-amylase were shown to be in the range from 0.1 to 0.7 mg/mL, and for α-glucosidase, from 0.1 to 0.2 mg/mL. In our studies, the Pearson coefficient showed a correlation for the skin between α-amylase and anthocyanin content (r = −0.91; *p* < 0.05) and a weaker one between α-glucosidase and anthocyanins (r = −0.81; *p* < 0.05) (Table S3).

### 2.6. Anti-Inflammatory Activity

Anti-inflammatory activity was assessed by measuring the ability to inhibit lipoxygenase (LOXI) and xanthine oxidase (XOI) activities (Table 5 and Table S2). There are no reports in the literature on the activity of haskap berries against these enzymes. Generally, significantly higher anti-inflammatory activity was demonstrated for the skin than for the flesh of haskap berry. For the skin, the IC_50_ values for LOXI ranged from 0.30 to 0.79 mg/mL and for XOI, from 1.11 to 2.12 mg/mL. The highest activity was exhibited by the Boreal Beast (LOXI) and Honeybee (XOI) cultivars. For the flesh of the same cultivars, the ability to inhibit LOXI and XOI activities was 3.9 and 2.2 times lower, respectively. These demonstrated activities, according to the Pearson correlation results, can be attributed to the content of polyphenolic compounds in the skin, mainly anthocyanins (r = −0.93 (LOXI); r = −0.82 (XOI); *p* < 0.05) (Table S3). A different view was presented by Rupasinghe et al. [32], who showed a negative correlation between all groups of polyphenolic compounds and the inhibition of the activity of proinflammatory cytokines TNF-α, IL-6 and COX-2. Moreover, in the cited work, the anti-inflammatory effect of extracts from haskap berry fruits (at a concentration of 100 μg/mL) was comparable to the effect of diclofenac. The anti-inflammatory activity of haskap berry fruits was also confirmed in in vivo studies. For mice stimulated with lipopolysaccharide, reducing the concentration of tumor necrosis factor (TNF-α), interleukin (IL)-10 and monocyte chemotactic protein-1 (MCP-1) [33] has been associated with two main components dominant in the extract—cyanidin 3-*O*-glycoside and (-)-epicatechin. In our study, we also did not find a significant effect of iridoid content on anti-inflammatory activity (r = −0.13 (LOXI); r = −0.25 (XOI); *p* < 0.05). Other authors reported that iridoids isolated from *Morinda officinalis* possess the ability to alleviate inflammation by binding to COX-2 and iNOS in in vitro studies [34], while in in vivo studies, they reduce the levels of interleukins IL-1β, IL-6 and IL-17 in serum in rats with induced inflammation [35]. Further studies are therefore indicated, including in vivo studies in particular, which will clearly indicate the active components of haskap fruits responsible for their anti-inflammatory effects.

### 2.7. Cytotoxic Activity Against Cancer Cells

The cytotoxic activity of the haskap berry fruit skin against cancer cell lines can be arranged in the following order: Caco-2 > AGS > Ht-29 > U87 mg > U251 mg > SK-Mel-29. In contrast, that of the fruit flesh is in the following order: Ht-29 > AGS > Caco-2 > U87 > SK-Mel-29 > U251 mg (Table 6 and Table S2). A significantly higher (*p* < 0.05) activity was found for the haskap berry skin, mainly that from the cv. ‘Honeybee’. The highest sensitivity to the extracts tested was characteristic of the cancer cell lines of the digestive tract, such as the colon and stomach. The obtained IC_50_ values for the fruit skin of the most active cultivar were 96.04 µg/mL (Ht-29 line) and 107.30 µg/mL (Caco-2 line) and 155.03 µg/mL (AGS line), respectively. On the other hand, the lowest sensitivity, regardless of the analyzed fruit part and cultivar, was demonstrated for the Sk-Mel-29 and U251 mg lines. Melanoma and astrocytoma cell lines are characterized by high therapeutic resistance. Hence, the low sensitivity of these lines to the tested raw material can be explained.

So far, the cytotoxic activity of haskap berry has been most widely analyzed against liver cancer cells (lines HepG2, SMMC-7721) [6,36,37] and prostate cancer cells (lines PC-3, LNCaP, C4-2, DU 145) [5]. Similar results were obtained against prostate cancer cells [5]. For the hexane extract of the fruit, values ranging from 89.6 (line DU145) to 140.8 (line LNCaP) μg/mL were shown. In turn, Zhou et al. [37], for purified anthocyanins from haskap berry fruits, proved anticancer properties against SMMC-7721 cells by blocking the cell cycle in the G2/M phase. The authors also proved in vivo inhibition of tumor growth in mice, regulating the level of immune cytokines and improving survival status. Also, Luo et al. [36], for three isolated anthocyanins from haskap berry fruits, showed the highest activity after treatment of SMMC-7721 cells with cyanidin 3-*O*-glucoside (10–110 μmol/mL). It was reported that the isolated compound caused cell apoptosis. In our study, the cytotoxic activity of the skin against Ht-29, Caco-2 and AGS lines also significantly correlated with the anthocyanin content (r = −0.88 (Ht-29); r = −0.87 (Caco-2); r = −0.81 (AGS); *p* < 0.05) (Table S3).

### 2.8. Antimicrobial Activity

Ethanol extracts from both the fruit skin and the flesh of all tested haskap berry cultivars at a dose of 100 mg/mL showed efficacy against Gram-positive bacteria, including *L. monocytogenes*, *E. faecalis* and *S. aureus* (Figure S1). However, no activity was indicated against *E. coli*, *S. enterica* sv. *Enteretidis*, *P. aeruginosa* and *C. albicans* strains. Also, a lack of antimicrobial activity was observed at lower concentrations (i.e. 1 and 10 mg/mL) for both aqueous and ethanol extracts. We speculate that the observed bactericidal effects at higher extract concentrations may be limited by the bioavailability of active ingredients to bacterial cells when the spot-on-lawn method is used (CFU/mL). On solid media, cells grow in clusters (colonies), which can prevent bioactive compounds from penetrating deep into the bacterial colony. This contrasts with free-floating cells of the same bacterial strains in liquid cultures. Similar results were obtained with aqueous and alcoholic extracts of *Planktochlorella nurekis* at concentrations of 100 mg/mL and 10 mg/mL when tested using the spot-on-lawn method. However, these extracts maintained their bactericidal effect in liquid culture at lower concentrations (i.e. 1 mg/mL, 100 µg/mL and 10 µg/mL respectively) [38]. The higher efficacy of ethanol extracts compared to aqueous extracts can be attributed to the increased solubility of lipophilic compounds, such as phenolic compounds, in ethanol. The bactericidal effect of dry extracts of haskap berries against *E. coli*, *L. monocytogenes*, *S. typhimurium* (3.41 mg/mL) and *S. aureus* and *B. cereus* (6.81 mg/mL) strains was previously confirmed [26]. Also, the team Česonienė et al. [25] presented the ability of haskap berry extracts to inhibit *E. faecalis* cultures; however, as in our study, this effect was higher for ethanol extracts than for aqueous extracts (inhibition zones 21.4 and 18.5 mm, respectively). The above results suggest that a certain threshold concentration of bioactive compounds is required to induce an antimicrobial effect. Polyphenolic compounds are known for their strong antioxidant and antimicrobial properties, which can disrupt bacterial cell membranes and interfere with essential metabolic processes [25]. This finding highlights the importance of optimizing the extraction process to obtain high content of bioactive compounds. Taken together, these findings demonstrate the potential of haskap berry extracts as an economical alternative source of antimicrobial agents, particularly in light of the growing problem of drug resistance among microorganisms.

### 2.9. Principal Component Analysis

**Figure 1:**
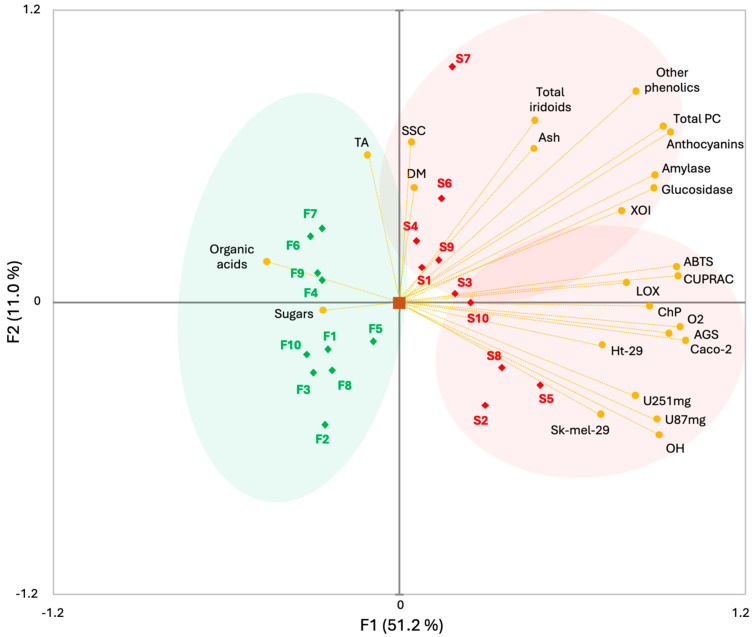
Principal component analysis of data for the physicochemical, health-promoting properties and bioactive composition of the fruit skin and flesh of 10 haskap berry cultivars. Explanations: S, skin; F, flesh; 1, Boreal Beauty; 2, Boreal Beast; 3, Boreal Blizzard; 4, Aurora; 5, Honeybee; 6, Vostarg; 7, Jugana; 8, Usłada; 9, Lawina; 10, Sinij Uties.

The tool used to visualize the relationship between the analyzed parameters and the studied samples was principal component analysis (PCA). The two principal components PC1 and PC2 explained 62.2% of the total variability of the data (Figure 1). At first glance, it can be seen that the peel and flesh of the haskap berry were separated on the opposite sides, which indicates that the morphological part had a significant effect on the values of the analyzed parameters. The PCA analysis distinguished three main clusters. Cluster 1 included the skin of the fruit of the Boreal Beauty, Boreal Blizzard, Aurora, Vostarg, Jugana, and Lawina cultivars, which significantly correlated with the polyphenolic and iridoid composition, as well as anti-inflammatory and antidiabetic activity. The second cluster was composed of the skin of the Boreal Beast, Honeybee, Usłada, and Sinij Uties cultivars, which dominated in terms of antioxidant and antiproliferative activity. The third group was represented by the fruit flesh of 10 tested cultivars, which were characterized by high TA, sugar and organic acid content. PCA results indicate variables with the greatest influence on the separation of samples in the PC1-PC2 space. Biochemical features, such as polyphenol and iridoid content and sugar and acid levels, proved to be crucial for the observed division between the two main groups of samples, emphasizing the metabolic diversity of fruit tissues and their potential functional significance. The obtained results are important from the point of view of designing new functional food additives or dietary supplements.

## 3. Materials and Methods

### 3.1. Reagents

Standards used for HPLC and UPLC analysis were purchased from Extrasynthese (Lyon, France) and Sigma-Aldrich (Darmstadt, Germany). α-Amylase from porcine pancreas, α-glucosidase from S. cerevisiae (type I), 2-Deoxy-D-ribose, 2.2-azinobis-3-ethylbenzthiazoline-6-sulphonic acid (ABTS), EDTA, ferrozine, LiChroprep RP-18 (40–63 µm), neocuproine, and trolox (6-hydroxy-2,5,7,8-tetramethylchroman-2-carboxylic acid) were purchased from Sigma-Aldrich (Steinheim, Germany). Cell lines Caco-2, Ht-29, and AGS were purchased from the Sigma-Aldrich company (ECACC, Steinheim, Germany). The other cell line was a gift from Prof. Jolanta Redowicz (Nencki Institute of Experimental Biology PAS, Warsaw, Poland). CellTiter 96^®^ AQueous Non-Radioactive Cell Proliferation Assay was purchased from Promega (Madison, WI, USA).

### 3.2. Plant Material

The study used the fruit skin and flesh of 10 haskap cultivars, including five Canadian cultivars: ‘Boreal Beauty’, ‘Boreal Beast’, ‘Boreal Blizzard’, ‘Aurora’, and ‘Honeybee’; and five Russian cultivars: ‘Vostarg’, ‘Jugana’, ‘Usłada’, ‘Lawina’, and ‘Sinij Uties’. The fruits were collected from a commercial field in Muniakowice, Małopolska Province, Poland (50°17′31.0″ N 20°06′59.0″ E). The fully ripe fruit samples of 3 kg of each cultivar were hand-picked from 5-year-old bushes from June to July 2021. After harvest, the fruits were transported to the laboratory, and the skin and flesh were manually separated, then frozen, freeze-dried (ALPHA 1-2 LD plus, Martin Christ GmbH, Osterode am Harz, Germany), and ground using a coffee grinder. The powders were stored in a refrigerator (−40 °C) until analysis.

### 3.3. Analysis of Physicochemical Properties

The soluble solids content (SSC) was determined using a refractometer (Pal-1 type, Conbest, Kraków, Poland), titratable acidity (TA) according to the PN-EN 12147:2000 [39] standard, dry matter (DM) according to the PN-A-75101-03:1990 standard [40], and total ash content according to the PN-90/A-75101/08 standard [41]. The determinations were made on fresh mass of raw material.

### 3.4. Analysis of Sugars and Organic Acids by HPLC

Sample extraction for HPLC analysis was performed as described by Česonienė et al. [25]. Analysis of sugars and organic acids was performed using a Sykam chromatograph (Sykam GmbH, Eresing, Germany) equipped with an S3590 RI detector, an S5250 sample injector, an S1125 pump system and an S4120 column oven. Separation was performed using a Eurokrat H 10 μm, 300 × 8 mm KNAUER GmbH (Berlin, Germany) column maintained at 90 °C. The flow rate was 0.5 mL/min. The injection volume was 20 μL. The mobile phase was 1.5 mM sulfuric acid in water in isocratic mode. Results are expressed in g/100 g d.m.

### 3.5. Analysis of Iridoids and Polyphenolic Compounds by UPLC-PDA-MS/MS

Extractions were used to analyze the content of iridoids and polyphenols, as well as to assess antioxidant, anti-inflammatory, and antidiabetic activity according to the protocol described earlier [11,42]. Briefly, powdered samples were combined 1:10 with 50% ethanol acidified with 0.1% formic acid. The extracts were then placed in an ultrasonic bath (Sonic 10, Polsonic, Warsaw, Poland) at 25 °C for 20 min, centrifuged at 7380 rpm for 15 min (5430, Eppendorf, Hamburg, Germany) and used for analyses.

Analysis of iridoids and polyphenolic compounds was performed using the Waters ACQUITY Ultra Performance Liquid Chromatography (UPLC) system (Waters, Milford, MA, USA), equipped with column manager, sample manager, binary pump manager, tandem quadrupole mass spectrometer (TQD) with electrospray ionization (ESI) source and photodiode array detector (PDA) and UPLC BEH C18 column (1.7 µm, 100 mm × 2.1 mm, Waters, Warsaw, Poland). The measurement was performed as described [11,12,43]. Separation was performed at 50 °C with an injection volume of 5 µL and a flow rate of 0.35 mL/min. The mobile phase for anthocyanin determination was 2% formic acid in water (solvent A) and 2% formic acid in 40% acetonitrile (solvent B), while for the remaining phenolic compounds, water (solvent A) and 40% acetonitrile (solvent B) were used. Mass spectrometer settings were as follows: desolvation temperature 350 °C, source block temperature 120 °C, cone voltage 30 V, capillary voltage 3.5 kV, desolvation gas flow rate 800 l/h. The obtained data were processed using MassLynx v.4.1 (Waters, Milford, MA, USA). Compounds were identified based on retention time, molecular weight, UV–Vis spectrum, MS/MS ions and literature data. Iridoids were monitored at 245 nm, flavan-3-ols at 280 nm, phenolic acids at 320 nm, flavones at 340 nm, flavonols glycosides at 360 nm, and anthocyanins at 520 nm. Results are expressed as mg/g d.m.

### 3.6. Antioxidant Activity Assay

Antioxidant activity was determined by the synthetic ABTS (ABTS) scavenging method described by Re et al. [44]. The extract was mixed with ABTS solution (diluted with distillated water to an absorbance of 0.7), and left for 6 min. The absorbance was measured at the wavelength of 734 nm.

The CUPRAC test was determined by the method described by Apak et al. [45]. The extract was mixed with 1.0 mL neocuproine (7.5 mM), acetate buffer (1 M, pH 7.0), and 1.0 mL copper chloride (10 mM), and left for 30 min. The absorbance was measured at the wavelength of 450 nm.

The ability of the extract to chelate iron ions (ChP) was assessed according to the method described by Żurek et al. [46]. The extract was mixed with 0.2 mL of iron II sulfate (0.1 mM) and 0.4 mL ferrozine solution (0.25 mM), and left for 10 min. The absorbance was measured at the wavelength of 562 nm.

Superoxide radical scavenging activity (O_2_˙^−^) was measured based on the method described by Żurek et al. [46]. The extract was mixed with 1.0 mL NBT (150 µM), 1.0 mL NADH (468 µM), and 1.0 mL PMS (60 µM), and left for 5 min. The absorbance was measured at the wavelength of 560 nm.

Hydroxyl radical scavenging activity (OH˙) was evaluated by the method of Żurek et al. [47]. The extract was mixed with a 0.9 mL mixture of 2-deoxyribose (0.2 mM), iron ammonium sulphate (1.0 mM), EDTA (1.04 mM), ascorbic acid (1.0 mM) and perhydrol (0.1 M). The solution was kept for 1 h at 37 °C, then was mixed with 1.0 mL trichloroacetic acid (2.8%) and 0.5 mL thiobarbituric acid (1%,). The absorbance was measured at the wavelength of 532 nm.

The absorbance was measured using a UV–Vis spectrometer (UV2900, Hitachi, Japan). The results for the ABTS and CUPRAC tests were expressed as Trolox Equivalent (mmol TE/100 g d.m.). However, results for the ChP, O_2_˙^−^ and OH˙ methods were expressed as the IC_50_ (μg/mL). Analyses for these three parameters were performed in the concentration range of 0.1–2.0 mg/mL. Ascorbic acid, EDTA and quercetin were used as positive controls.

### 3.7. Antidiabetic Activity Assay

The inhibitory activity of α-amylase was measured using Red-starch as a substrate. The reaction mixture contained 120 μL of 50 mmol/L phosphate buffer pH 6.6 containing 6 mmol/L NaCl, 10 μL of the enzyme (10 ug/mL, α-amylase from porcine pancreas, 50 U/mg) and 20 μL of the substrate (2%). The reaction mixture was incubated for 20 min at 40 °C and stopped by adding 150 μL of pure ethanol. After centrifugation (15 min, 25 °C, 5000× *g*), 150 μL was transferred into new plates, and absorbance was measured at 510 nm. For the inhibition studies, before adding the substrate, the enzyme was incubated for 10 min with 20 μL of the studied extract.

α-Glucosidase inhibitor activity was measured according to the method described by Lachowicz et al. [48]. Briefly, 10 μL of α-glucosidase (1 U/mL) and 20 μL of 1% sucrose were added to 0.5 mL of 0.1 mol/L phosphate buffer, pH 6.8. The reaction was incubated at 37 °C for 5 min, stopped by the addition of 100 μL of 3,5-dinitrosalicylic acid (DNS) and heated for 10 min. Then, the mixture was made up to 300 μL with double-distilled water, and the absorbance was measured at 540 nm. For αGIA measurement, 10 μL of α-glucosidase (1 U/mL) and 50 μL of sample were added to 0.45 mL of 0.1 mol/l phosphate buffer, pH 6.8. After incubation at 37 °C for 5 min, 20 μL of 1% sucrose was added. The reaction was incubated at 37 °C for 50 min, stopped by the addition of 100 μL of 3,5-dinitrosalicylic acid (DNS) and heated for 10 min. Absorbance was measured at 540 nm.

Results for both methods are expressed as IC_50_. Analyses were performed in the concentration range of 1.0–50.0 mg/mL. Acarbose was used as a positive control.

### 3.8. Anti-Inflammatory Activity Assay

The inhibitory activity of lipoxygenase (LOXI) was measured using linoleic acid as a substrate. The reaction mixture contained 250 μL of 1/15 M sodium-phosphate buffer, 10 μl of the enzyme (10 μg/mL) and 40 μL of the substrate (5 mmol). The change in absorbance (3 min) was measured at 252 nm. For the inhibition studies, before adding the substrate, the enzyme was incubated for 10 min with 10 μL of the studied extract.

The inhibitory activity of Xanthine oxidase (XOI) was measured using xanthine as a substrate. The reaction mixture contained 120 μL of 1/15 M sodium-phosphate buffer, 20 μL of the enzyme xanthine oxidase (10 μL/mL) and 20 μL of the substrate (0.015 mmol). The change in absorbance (3 min) was measured at 234 nm. For the inhibition studies, before adding the substrate, the enzyme was incubated for 10 min with 20 μL of the studied extract.

All inhibitory assays were read using a BioTek Epoch microplate reader. The results were expressed as IC_50_ (mg/mL). Analyses were performed in the concentration range of 1.0–50.0 mg/mL. Allopurynol and quercetin were used as a positive control.

### 3.9. Assessment of Cytotoxic Activity Against Cancer Cells

To assess cell viability, the extracts were additionally extracted into the solid phase using the LiChroprep RP-18 adsorber [49]. Six cancer cell lines were selected for the study: colon adenocarcinoma (Caco-2, HT-29), astrocytoma (U251 mg), glioblastoma (U87 mg), gastric adenocarcinoma (AGS) and melanoma (SK-Mel-29). The cells were cultured in DMEM and McCoy’s 5A medium supplemented with 10% inactivated fetal serum and 1% antibiotic solution. The culture was carried out in an incubator (CB170, Binder, Tuttlinen, Germany) in a humidified atmosphere at 5% CO_2_ at 37 °C. After the fourth passage, the cells were seeded on 96-well plates (8 × 10^3^ cells/well) and allowed to adhere. After 24 h, the cells were treated for 48 h with haskap berry preparations. Cytotoxicity was assessed using the MTS assay (Promega). Absorbance was measured at 490 nm on a microplate reader (SmartReader 96, Accuris Instruments. Edison, NJ, USA). Results were expressed as IC_50_ (µg/mL). Cisplatin was used as a positive control.

### 3.10. Assessment of Antimicrobial Activity Against Selected Microorganisms

To assess antimicrobial activity, aqueous extracts were prepared by boiling at 100 °C for 30 min, and ethanol extracts (80% ethanol) were incubated at 37 °C for 24 h with shaking at 5000 rpm. After centrifugation, the supernatants were used for analyses. The antimicrobial potency was determined using the following microorganisms: three Gram-negative strains (*Escherichia coli* PCM 2209, *Salmonella enterica* sv. *Enteretidis*, *Pseudomonas aeruginosa* DSM 19880) and three Gram-positive strains (*Staphylococcus aureus* PCM 458, *Listeria monocytogenes* PCM 2191 and *Enterococcus faecalis*). Additionally, the antifungal activity of the tested extracts was checked using *Candida albicans* ATCC 14053 strain. Clinical strains *Salmonella enterica* sv. *enteretidis* and *Enterococcus faecalis* were received from the collections of microorganisms of the Frederic Chopin Provincial Specialist Hospital in Rzeszów, Poland. The microorganisms were cultured on Nutrient Agar NA and on YPD medium. The antimicrobial activity of aqueous and ethanolic haskap berry extracts (1:10 and 1:100 dilutions) was evaluated using a spot-on-lawn assay against selected microorganisms (8-log CFU/mL). Ethanol dilutions (80%, 8%, 0.8%) were used as positive controls.

Growth inhibition zones were measured after 24 h (bacteria at 37 °C) or 29 °C (yeast) incubation and recorded using a Canon EOS 600D camera (Canon Inc., Warsaw, Poland).

### 3.11. Statistical Analysis

The results in tables and graphs are presented as mean and standard deviation (SD). All statistical analyses (Duncan test, Pearson correlation, PCA) were performed using Statistica software version 13.3 (StatSoft, Kraków, Poland).

## 4. Conclusions

This study is the first comprehensive report on the bioactive composition, physicochemical and health-promoting properties of the skin and flesh isolated from haskap berry fruits. The results of the conducted analyses indicated the occurrence of statistically significant differences between the analyzed parameters and the fruit parts and their cultivars. The fruit skin contained mainly anthocyanins, phenolic acids and iridoids and was also characterized by high antioxidant, anti-inflammatory, antidiabetic and anti-proliferation activity, especially against colon and stomach cancer cells. The skin of the cv. ‘Honeybee’ was particularly distinguished in terms of these features. In turn, quercetin derivatives, sugars and organic acids were mainly located in the fruit flesh of the studied cultivars, including the highest concentrations in the cvs. ‘Honeybee’, ‘Vostorg’ and ‘Usłada’. In the case of antibacterial activity, no differences were shown between the studied fruit parts or cultivars. The results of the study indicate that the peel of the haskap berry is a good material for further analysis, including the isolation of polyphenolic compounds and iridoids, which can be used in the pharmaceutical, cosmetic and food industries, including the production of dietary supplements and functional foods supporting the treatment and prevention of type 2 diabetes, colon cancer, inflammation or chronic diseases resulting from disturbances in the body’s oxidative balance. An interesting solution would also be to determine the optimal proportions of skin to flesh based on bioactivity thresholds, which would allow for the formulation of practical guidelines for the design of effective nutraceutical agents. However, further studies are necessary, including in vivo and clinical studies, to finally confirm their effectiveness.

## Figures and Tables

**Table 1:** Physicochemical parameters of the fruit skin and flesh of 10 cultivars of haskap berries.

Cultivars and Parts	SSC	TA	DM	Ash
	°Brix	g/100 g f.m.	g/100 g f.m.	%
Skin				
Boreal Beauty	13.51 ± 0.07 ^e^	3.01 ± 0.02 ^d^	14.01 ± 0.07 ^bc^	0.55 ± 0.00 ^b^
Boreal Beast	14.32 ± 0.06 ^gh^	2.22 ± 0.01 ^a^	13.82 ± 0.05 ^b^	0.53 ± 0.00 ^a^
Boreal Blizzard	13.80 ± 0.08 ^ef^	2.41 ± 0.01 ^ab^	15.58 ± 0.01 ^f^	0.57 ± 0.01 ^b^
Aurora	15.71 ± 0.04 ^ij^	2.89 ± 0.01 ^cd^	15.41 ± 0.05 ^ef^	0.51 ± 0.00 ^a^
Honeybee	14.07 ± 0.06 ^g^	3.11 ± 0.06 ^e^	17.20 ± 0.05 ^i^	0.55 ± 0.00 ^b^
Vostorg	12.68 ± 0.05 ^cd^	3.18 ± 0.03 ^f^	16.03 ± 0.06 ^g^	0.60 ± 0.00 ^b^
Jugana	13.71 ± 0.07 ^e^	3.12 ± 0.04 ^e^	15.41 ± 0.0.6 ^ef^	0.61 ± 0.00 ^b^
Usłada	16.04 ± 0.16 ^j^	2.58 ± 0.07 ^b^	14.37 ± 0.15 ^d^	0.63 ± 0.01 ^b^
Lawina	14.47 ± 0.09 ^h^	2.81 ± 0.05 ^cd^	14.18 ± 0.05 ^c^	0.67 ± 0.00 ^c^
Sinij Uties	15.46 ±0.11 ^i^	2.89 ± 0.02 ^cd^	15.20 ± 0.06 ^ef^	0.56 ± 0.00 ^b^
Flesh				
Boreal Beauty	12.13 ± 0.11 ^c^	2.84 ± 0.02 ^cd^	13.54 ± 0.12 ^a^	0.46 ± 0.00 ^a^
Boreal Beast	10.68 ± 0.09 ^ab^	2.06 ± 0.01 ^a^	13.51 ± 0.05 ^a^	0.50 ± 0.01 ^a^
Boreal Blizzard	10.22 ± 0.10 ^a^	2.32 ± 0.02 ^ab^	15.34 ± 0.07 ^ef^	0.51 ± 0.00 ^a^
Aurora	12.10 ± 0.04 ^c^	2.78 ± 0.01 ^cd^	15.22 ± 0.06 ^ef^	0.49 ± 0.00 ^a^
Honeybee	12.07 ± 0.04 ^c^	2.94 ± 0.05 ^cd^	16.84 ± 0.10 ^h^	0.52 ± 0.00 ^a^
Vostorg	11.23 ± 0.06 ^b^	3.10 ± 0.05 ^e^	15.88 ± 0.11 ^g^	0.58 ± 0.01 ^b^
Jugana	13.01 ± 0.11 ^d^	2.86 ± 0.02 ^cd^	15.07 ± 0.07 ^e^	0.58 ± 0.00 ^b^
Usłada	14.22 ± 0.12 ^g^	2.53 ± 0.02 ^b^	14.18 ± 0.08 ^c^	0.60 ± 0.00 ^b^
Lawina	14.01 ± 0.02 ^f^	2.63 ± 0.04 ^b^	14.11 ± 0.11 ^c^	0.61 ± 0.00 ^b^
Sinij Uties	13.46 ± 0.07 ^e^	2.69 ± 0.04 ^c^	14.98 ± 0.14 ^e^	0.50 ± 0.00 ^a^
Mean				
Skin	14.33	2.81	15.12	0.58
Flesh	12.31	2.68	14.87	0.54

Values are mean (*n* = 3) and SD. Values followed by different letters (a–j) in the same column indicate significant differences (*p* < 0.05).

**Table 2:** Content of polyphenolic compounds in the fruit skin and flesh of 10 cultivars of haskap berries.

**Cultivars and Parts**	**Anthocyanins (mg/g d.m.)**											
	**(Epi)catechin-Cyanidin 3-*O*-glucoside**	**Cyanidin 3-*O*-sophoroside-5-*O*-glucoside**		**Cyanidin 3-*O*-rutinoside-5-*O*-glucoside**	**Cyanidin 3,5-*O*-diglucoside**	**Cyanidin 3-*O*-glucoside**	**Cyanidin 3-*O*-rutinoside**	**Pelargonidin 3-*O*-glucoside**	**Peonidin 3-*O*-glucoside**	**Peonidin 3-*O*-rutinoside**	**Delphinidin 3-*O*-rhamnoside**	**Sum Anthocyanins**
Skin												
Boreal Beauty	<LLQ	<LLQ		<LLQ	1.23 ± 0.01 ^e^	51.48 ± 0.50 ^f^	4.59 ± 0.41 ^i^	0.60 ± 0.03 ^e^	2.25 ± 0.04 ^h^	0.65 ± 0.04 ^e^	<LLQ	61.71 ± 3.24 ^h^
Boreal Beast	<LLQ	<LLQ		<LLQ	4.18 ± 0.30 ^j^	63.59 ± 0.44 ^j^	5.03 ± 0.31 ^j^	0.58 ± 0.03 ^e^	2.32 ± 0.02 ^h^	0.59 ± 0.00 ^d^	<LLQ	77.50 ± 1.01 ^m^
Boreal Blizzard	<LLQ	<LLQ		<LLQ	2.98 ± 0.20 ^h^	64.17 ± 0.33 ^k^	4.19 ± 0.13 ^h^	0.60 ± 0.00 ^e^	2.98 ± 0.11 ^m^	0.60 ± 0.02 ^d^	<LLQ	75.49 ± 0.74 ^k^
Aurora	<LLQ	<LLQ		<LLQ	1.39 ± 0.04 ^f^	56.29 ± 0.74 ^g^	2.04 ± 0.02 ^de^	0.65 ± 0.04 ^f^	2. 52 ± 0.11 ^j^	0.45 ± 0.03 ^c^	<LLQ	64.33 ± 2.13 ^i^
Honeybee	<LLQ	<LLQ		<LLQ	3.44 ± 0.31 ^i^	82.34 ± 0.30 ^m^	7.66 ± 0.24 ^n^	0.80 ± 0.01 ^g^	2.73 ± 0.11 ^k^	0.64 ± 0.03 ^d^	<LLQ	98.50 ± 1.91 ^n^
Vostorg	<LLQ	<LLQ		<LLQ	1.48 ± 0.03 ^fg^	64.68 ± 0.93 ^l^	5.49 ± 0.03 ^k^	0.47 ± 0.02 ^d^	2.97 ± 0.23 ^l^	0.64 ± 0.01 ^d^	<LLQ	76.44 ± 1.03 ^l^
Jugana	<LLQ	<LLQ		<LLQ	2.86 ± 0.11 ^h^	51.58 ± 0.54 ^f^	2.77 ± 0.04 ^g^	0.45 ± 0.01 ^d^	1.69 ± 0.03 ^f^	0.48 ± 0.00 ^c^	<LLQ	60.39 ± 1.59 ^g^
Usłada	<LLQ	<LLQ		<LLQ	1.60 ± 0.01 ^g^	58.56 ± 0.31 ^h^	7.72 ± 0.22 ^n^	0.56 ± 0.00 ^e^	2.06 ± 0.04 ^g^	0.70 ± 0.00 ^e^	<LLQ	71.88 ± 0.73 ^j^
Lawina	<LLQ	<LLQ		<LLQ	1.46 ± 0.04 ^fg^	59.38 ± 0.38 ^i^	5.96 ± 0.13 ^l^	0.83 ± 0.03 ^g^	2.38 ± 0.11 ^hi^	0.66 ± 0.00 ^e^	<LLQ	71.78 ± 0.77 ^j^
Sinij Uties	<LLQ	<LLQ		<LLQ	1.63 ± 0.03 ^g^	63.30 ± 0.83 ^j^	6.83 ± 0.33 ^m^	0.93 ± 0.01 ^h^	2.74 ± 0.22 ^k^	0.71 ± 0.00 ^e^	<LLQ	76.61 ± 1.20 ^l^
Flesh												
Boreal Beauty	<LLQ	<LLQ		<LLQ	0.32 ± 0.00 ^ab^	17.57 ± 0.17 ^b^	2.44 ± 0.04 ^f^	0.13 ± 0.00 ^a^	0.96 ± 0.01 ^c^	0.22 ± 0.02 ^b^	<LLQ	22.03 ± 1.08 ^c^
Boreal Beast	<LLQ	<LLQ		<LLQ	0.61 ± 0.00 ^c^	16.97 ± 0.05 ^b^	1.60 ± 0.03 ^c^	0.14 ± 0.01 ^a^	0.69 ± 0.03 ^ab^	0.14 ± 0.00 ^a^	<LLQ	20.14 ± 0.41 ^b^
Boreal Blizzard	<LLQ	<LLQ		<LLQ	0.88 ± 0.03 ^d^	20.41 ± 0.18 ^de^	1.93 ± 0.03 ^d^	0.18 ± 0.00 ^b^	1.32 ± 0.02 ^e^	0.17 ± 0.00 ^b^	<LLQ	24.91 ± 1.88 ^ef^
Aurora	<LLQ	<LLQ		<LLQ	0.37 ± 0.02 ^b^	20.86 ± 0.21 ^e^	0.90 ± 0.01 ^a^	0.24 ± 0.02 ^b^	1.27 ± 0.03 ^e^	<LLQ	<LLQ	24.01 ± 0.77 ^e^
Honeybee	<LLQ	<LLQ		<LLQ	0.80 ± 0.10 ^d^	16.20 ± 0.11 ^a^	1.21 ± 0.02 ^b^	<LLQ	0.64 ± 0.02 ^a^	<LLQ	<LLQ	19.04 ± 1.07 ^a^
Vostorg	<LLQ	<LLQ		<LLQ	0.19 ± 0.01 ^a^	17.25 ± 0.32 ^b^	1.54 ± 0.03 ^c^	<LLQ	0.90 ± 0.01 ^c^	<LLQ	<LLQ	19.66 ± 0.70 ^ab^
Jugana	<LLQ	<LLQ		<LLQ	0.70 ± 0.01 ^cd^	21.01 ± 0.42 ^e^	2.14 ± 0.13 ^e^	0.17 ± 0.00 ^b^	0.81 ± 0.02 ^b^	0.08 ± 0.00 ^a^	<LLQ	25.08 ± 1.52 ^f^
Usłada	<LLQ	<LLQ		<LLQ	0.31 ± 0.00 ^ab^	17.43 ± 0.11 ^b^	2.73 ± 0.14 ^g^	0.15 ± 0.00 ^b^	0.78 ± 0.03 ^b^	0.20 ± 0.00 ^b^	<LLQ	21.77 ± 0.32 ^c^
Lawina	<LLQ	<LLQ		<LLQ	0.40 ± 0.00 ^b^	19.85 ± 0.18 ^d^	2.84 ± 0.07 ^g^	0.29 ± 0.00 ^c^	1.13 ± 0.01 ^d^	0.21 ± 0.00 ^b^	<LLQ	25.10 ± 0.77 ^f^
Sinij Uties	<LLQ	<LLQ		<LLQ	0.39 ± 0.00 ^b^	18.95 ± 0.47 ^c^	2.45 ± 0.10 ^f^	0.32 ± 0.00 ^c^	1.04 ± 0.00 ^c^	0.20 ± 0.00 ^b^	<LLQ	23.52 ± 0.35 ^d^
Mean												
Skin	0.00	0.00		0.00	2.22	61.54	5.23	0.65	2.46	0.61	0.00	73.46
Flesh	0.00	0.00		0.00	0.50	18.64	1.97	0.20	0.95	0.18	0.00	22.52
**Cultivars and Parts**	**Other Phenolics (mg/g d.m.)**											
	**Neochlorogenic** **Acid**	**Procyanidin dimer B-type**	**Chlorogenic Acid**	**Procyanidin Dimer B-type**		**Luteolin 7-*O*-glucoside**	**Quercetin 3-*O*-rutinoside-7-*O*-rhamnoside**	**Quercetin 3-*O*-pentoside-glucoside I**	**Quercetin 3-*O*-rutinoside**	**Quercetin 3-*O*-glucoside**	**Quercetin 3-*O*-rhmanoside**	
Skin												
Boreal Beauty	0.16 ± 0.00 ^b^	0.10 ± 0.00 ^a^	1.75 ± 0.20 ^d^	0.15 ± 0.00 ^b^		0.83 ± 0.00 ^c^	0.13 ± 0.00 ^a^	0.56 ± 0.01 ^e^	0.51 ± 0.03 ^b^	0.30 ± 0.00 ^b^	<LLQ	
Boreal Beast	0.27 ± 0.01 ^c^	<LLQ	1.74 ± 0.10 ^c^	0.16 ± 0.00 ^b^		1.09 ± 0.03 ^de^	0.18 ± 0.01 ^b^	0.15 ± 0.01 ^b^	1.90 ± 0.14 ^g^	0.21 ± 0.01 ^ab^	0.08 ± 0.00 ^a^	
Boreal Blizzard	0.11 ± 0.00 ^a^	0.06 ± 0.00 ^a^	1.93 ± 0.04 ^d^	0.15 ± 0.01 ^b^		1.16 ± 0.04 ^e^	0.09 ± 0.00 ^a^	0.18 ± 0.01 ^b^	1.21 ± 0.04 ^d^	0.19 ± 0.03 ^ab^	<LLQ	
Aurora	0.11 ± 0.00 ^a^	<LLQ	1.31 ± 0.03 ^a^	0.10 ± 0.00 ^a^		0.94 ± 0.01 ^c^	0.07 ± 0.00 ^a^	0.12 ± 0.00 ^a^	0.56 ± 0.03 ^c^	0.19 ± 0.01 ^ab^	<LLQ	
Honeybee	0.27 ± 0.03 ^c^	<LLQ	4.40 ± 0.41 ^i^	0.34 ± 0.01 ^c^		1.75 ± 0.04 ^f^	0.08 ± 0.00 ^a^	0.22 ± 0.00 ^b^	1.53 ± 0.10 ^e^	0.52 ± 0.00 ^c^	0.09 ± 0.00 ^a^	
Vostorg	0.27 ± 0.01 ^c^	<LLQ	2.16 ± 0.30 ^f^	0.19 ± 0.00 ^b^		1.15 ± 0.04 ^e^	0.14 ± 0.0 ^a^	0.41 ± 0.00 ^d^	1.66 ± 0.20 ^f^	0.26 ± 0.00 ^b^	<LLQ	
Jugana	0.26 ± 0.02 ^c^	<LLQ	1.58 ± 0.10 ^c^	0.13 ± 0.00 ^a^		0.92 ± 0.02 ^d^	<LLQ	0.19 ± 0.00 ^b^	0.28 ± 0.00 ^a^	0.21 ± 0.00 ^ab^	<LLQ	
Usłada	0.29 ± 0.01 ^c^	<LLQ	2.64 ± 0.08 ^g^	0.22 ± 0.02 ^b^		1.03 ± 0.04 ^d^	0.09 ± 0.00 ^a^	0.23 ± 0.00 ^b^	1.93 ± 0.04 ^g^	0.23 ± 0.01 ^ab^	<LLQ	
Lawina	0.24 ± 0.00 ^b^	<LLQ	2.69 ± 0.11 ^gh^	0.21 ± 0.00 ^b^		1.07 ± 0.04 ^de^	0.17 ± 0.00 ^b^	0.25 ± 0.00 ^c^	1.34 ± 0.00 ^d^	0.29 ± 0.00 ^b^	0.07 ± 0.00 ^a^	
Sinij Uties	0.25 ± 0.01 ^c^	<LLQ	2.84 ± 0.11 ^gh^	0.25 ± 0.01 ^c^		1.19 ± 0.04 ^e^	0.19 ± 0.01 ^b^	0.28 ± 0.02 ^c^	1.47 ± 0.04 ^e^	0.32 ± 0.03 ^b^	0.09 ± 0.00 ^a^	
Flesh												
Boreal Beauty	0.13 ± 0.00 ^a^	0.05 ± 0.00 ^a^	1.66 ± 0.17 ^c^	0.06 ± 0.00 ^a^		0.56 ± 0.04 ^ab^	0.09 ± 0.00 ^a^	0.40 ± 0.00 ^d^	0.38 ± 0.00 ^b^	0.22 ± 0.00 ^a^	0.05 ± 0.00 ^a^	
Boreal Beast	0.15 ± 0.00 ^b^	<LLQ	1.47 ± 0.11 ^b^	0.05 ± 0.00 ^a^		0.56 ± 0.02 ^ab^	0.06 ± 0.00 ^a^	0.05 ± 0.00 ^a^	0.46 ± 0.01 ^b^	0.08 ± 0.00 ^a^	<LLQ	
Boreal Blizzard	0.05 ± 0.00 ^a^	0.05 ± 0.00 ^a^	1.92 ± 0.15 ^d^	0.07 ± 0.00 ^a^		0.79 ± 0.03 ^c^	0.06 ± 0.00 ^a^	0.13 ± 0.00 ^a^	0.72 ± 0.02 ^c^	0.14 ± 0.00 ^a^	<LLQ	
Aurora	0.07 ± 0.00 ^a^	0.05 ± 0.00 ^a^	1.42 ± 0.06 ^ab^	<LLQ		0.72 ± 0.03 ^ab^	0.05 ± 0.00 ^a^	0.08 ± 0.00 ^a^	0.37 ± 0.04 ^b^	0.18 ±0.00 ^a^	<LLQ	
Honeybee	0.17 ± 0.01 ^b^	0.06 ± 0.00 ^a^	3.05 ± 0.04 ^h^	0.07 ± 0.00 ^a^		0.81 ± 0.00 ^c^	<LLQ	0.07 ± 0.00 ^a^	0.41 ± 0.00 ^b^	0.16 ± 0.00 ^a^	<LLQ	
Vostorg	0.13 ± 0.00 ^a^	<LLQ	1.60 ± 0.03 ^c^	<LLQ		0.54 ± 0.04 ^a^	<LLQ	0.11 ± 0.00 ^a^	0.37 ± 0.03 ^b^	0.09 ± 0.00 ^a^	<LLQ	
Jugana	0.17 ± 0.01 ^b^	0.05 ± 0.00 ^a^	1.57 ± 0.04 ^c^	0.06 ± 0.00 ^a^		0.50 ± 0.00 ^a^	<LLQ	0.15 ± 0.00 ^b^	0.23 ± 0.00 ^a^	0.17 ± 0.00 ^a^	<LLQ	
Usłada	0.20 ± 0.00 ^b^	<LLQ	2.28 ± 0.24 ^f^	0.06 ± 0.00 ^a^		0.62 ± 0.00 ^ab^	<LLQ	0.08 ± 0.00 ^a^	0.49 ± 0.02 ^b^	0.10 ± 0.00 ^a^	<LLQ	
Lawina	0.16 ± 0.00 ^b^	<LLQ	2.27 ± 0.17 ^f^	0.07 ± 0.00 ^a^		0.76 ± 0.02 ^c^	0.08 ± 0.00 ^a^	0.12 ± 0.00 ^a^	0.60 ± 0.03 ^c^	0.16 ± 0.02 ^a^	0.05 ± 0.00 ^a^	
Sinij Uties	0.14 ± 0.00 ^a^	<LLQ	2.14 ± 0.04 ^e^	0.06 ± 0.00 ^a^		0.68 ± 0.03 ^ab^	0.07 ± 0.00 ^a^	0.10 ± 0.00 ^a^	0.49 ±0.00 ^b^	0.14 ± 0.00 ^a^	<LLQ	
Mean												
Skin	0.22	0.02	2.30	0.19		1.11	0.12	0.26	1.24	0.26	0.07	
Flesh	0.14	0.04	1.94	0.06		0.65	0.06	0.13	0.45	0.14	0.03	
**Cultivars and Parts**	**Other phenolics** **(mg/g d.m.)**											
	**Kaempferol 3-*O*-glucoside-7-*O*-rhamnoside**	**Kaempferol 3-*O*-rutinoside**		**Kaempferol 3-O-glucoside-7-*O*-glucuronide**	**3,4-di-*O*-caffeoyl-quinic acid**	**Quercetin 3-*O*-(6”-acetylo)-glucoside**	**Kaempferol 3-*O*-rhamnoside**	**Kaempferol 3-*O*-pentoside I**	**Kaempferol 3-*O*-pentoside II**	**Quercetin 3-*O*-rhamnoside**	**Sum Other Phenolics**	**Total Polyphenols Compounds**
Skin												
Boreal Beauty	0.08 ± 0.00 ^a^	<LLQ		<LLQ	0.17 ± 0.00 ^b^	0.06 ± 0.00 ^a^	4.76 ± 0.31 ^d^	<LLQ	<LLQ	<LLQ	9.72 ± 0.51 ^f^	71.44 ± 3.70 ^k^
Boreal Beast	0.08 ± 0.00 ^a^	<LLQ		0.07 ± 0.00 ^a^	0.50 ± 0.00 ^a^	0.11 ± 0.00 ^a^	6.24 ± 0.24 ^f^	<LLQ	<LLQ	<LLQ	12.74 ± 0.63 ^i^	90.18 ± 4.50 ^o^
Boreal Blizzard	<LLQ	<LLQ		0.11 ± 0.00 ^a^	0.09 ± 0.00 ^a^	0.06 ± 0.00 ^a^	5.41 ± 0.21 ^e^	<LLQ	<LLQ	<LLQ	10.83 ± 0.81 ^g^	86.27 ± 2.19 ^m^
Aurora	<LLQ	0.06 ± 0.00 ^a^		0.05 ± 0.00 ^a^	0.11 ± 0.00 ^a^	0.07 ± 0.00 ^a^	3.70 ± 0.31 ^b^	<LLQ	<LLQ	<LLQ	7.50 ± 0.37 ^b^	71.41 ± 3.69 ^k^
Honeybee	<LLQ	<LLQ		0.11 ± 0.00 ^a^	0.15 ± 0.00 ^b^	0.09 ± 0.00 ^a^	9.64 ± 0.10 ^j^	<LLQ	<LLQ	<LLQ	19.48 ± 1.18 ^m^	118.01 ± 5.01 ^r^
Vostorg	0.05 ± 0.00 ^a^	<LLQ		0.11 ± 0.00 ^a^	0.11 ± 0.00 ^a^	0.10 ± 0.00 ^a^	6.69 ± 0.17 ^g^	<LLQ	<LLQ	<LLQ	13.51 ± 0.77 ^j^	89.91 ± 1.40 ^n^
Jugana	<LLQ	<LLQ		<LLQ	0.24 ± 0.02 ^b^	0.11 ± 0.00 ^a^	4.04 ± 0.03 ^c^	<LLQ	<LLQ	<LLQ	8.19 ± 0.46 ^c^	68.61 ± 2.01 ^j^
Usłada	<LLQ	0.08 ± 0.01 ^a^		0.11 ± 0.02 ^a^	0.08 ± 0.00 ^a^	<LLQ	7.06 ± 0.30 ^h^	<LLQ	<LLQ	<LLQ	14.32 ± 0.90 ^k^	86.09 ± 3.10 ^m^
Lawina	0.05 ± 0.00 ^a^	0.06 ± 0.00 ^a^		0.09 ± 0.00 ^a^	0.08 ± 0.00 ^a^	0.06 ± 0.00 ^a^	6.66 ± 0.18 ^g^	<LLQ	<LLQ	<LLQ	13.53 ± 0.17 ^j^	84.80 ± 1.19 ^l^
Sinij Uties	0.05 ± 0.00 ^a^	0.07 ± 0.00 ^a^		0.09 ± 0.00 ^a^	0.07 ± 0.00 ^a^	0.08 ± 0.00 ^a^	7.25 ± 0.21 ^i^	<LLQ	<LLQ	<LLQ	14.59 ± 0.20 ^l^	91.50 ± 3.08 ^p^
Flesh												
Boreal Beauty	0.05 ± 0.00 ^a^	<LLQ		<LLQ	0.40 ± 0.01 ^c^	0.05 ± 0.00 ^a^	<LLQ	<LLQ	<LLQ	4.08 ± 0.22 ^c^	8.34 ± 0.44 ^c^	29.80 ± 0.71 ^c^
Boreal Beast	<LLQ	<LLQ		<LLQ	0.58 ± 0.02 ^e^	0.05 ± 0.00 ^a^	<LLQ	<LLQ	<LLQ	3.63 ± 0.11 ^a^	7.41 ± 0.18 ^a^	27.77 ± 1.39 ^b^
Boreal Blizzard	<LLQ	<LLQ		<LLQ	0.39 ± 0.00 ^c^	0.05 ± 0.00 ^a^	<LLQ	<LLQ	<LLQ	4.41 ± 0.26 ^d^	8.81 ± 0.73 ^d^	33.69 ± 2.45 ^g^
Aurora	<LLQ	<LLQ		<LLQ	0.41 ± 0.04 ^c^	0.07 ± 0.00 ^a^	<LLQ	<LLQ	<LLQ	3.66 ± 0.18 ^b^	7.43 ± 0.22 ^a^	31.15 ± 1.88 ^e^
Honeybee	<LLQ	<LLQ		<LLQ	0.98 ± 0.07 ^g^	0.06 ± 0.00 ^a^	0.05 ± 0.00 ^a^	<LLQ	<LLQ	5.88 ± 0.09 ^g^	11.85 ± 0.66 ^h^	37.02 ± 0.77 ^i^
Vostorg	<LLQ	<LLQ		<LLQ	0.72 ± 0.01 ^f^	0.10 ± 0.00 ^a^	<LLQ	<LLQ	<LLQ	3.69 ± 0.22 ^b^	7.47 ± 0.35 ^b^	27.51 ± 1.67 ^b^
Jugana	0.05 ± 0.00 ^a^	<LLQ		<LLQ	0.47 ± 0.03 ^d^	0.10 ± 0.00 ^a^	<LLQ	<LLQ	0.05 ± 0.00 ^a^	3.59 ± 0.04 ^a^	7.33 ± 0.40 ^a^	26.31 ± 1.60 ^a^
Usłada	<LLQ	<LLQ		<LLQ	0.54 ± 0.02 ^d^	<LLQ	<LLQ	<LLQ	<LLQ	4.45 ± 0.11 ^de^	9.14 ± 0.48 ^e^	30.92 ± 0.8 ^d^
Lawina	<LLQ	0.05 ± 0.00 ^a^		<LLQ	0.51 ± 0.00 ^d^	0.05 ± 0.00 ^a^	<LLQ	<LLQ	<LLQ	4.90 ± 0.24 ^f^	9.74 ± 0.31 ^f^	34.81 ± 2.10 ^h^
Sinij Uties	<LLQ	<LLQ		<LLQ	0.56 ± 0.02 ^e^	0.06 ± 0.00 ^a^	<LLQ	<LLQ	<LLQ	4.56 ± 0.18 ^e^	9.21 ± 0.14 ^e^	32.77 ± 2.02 ^f^
Mean												
Skin	0.04	0.04		0.08	0.12	0.08	6.15	0.00	0.00	0.00	12.41	85.81
Flesh	0.00	0.00		0.00	0.55	0.06	0.00	0.00	0.00	4.29	8.65	31.18

Values are mean (*n* = 3) and SD. Values followed by different letters (a–r) in the same column indicate significant differences (*p* < 0.05). Legend: <LLQ, value below detection limit.

**Table 3:** Content of iridoid compounds in the fruit skin and flesh of 10 cultivars of haskap berries.

Cultivars and Parts	Iridoids (mg/g d.m.)						
	Loganic Acid	Sweroside Pentoside Isomer I	Loganin	Loganin Pentoside	Sweroside	Sweroside Pentoside Isomer II	Total Iridoids
Skin							
Boreal Beauty	3.66 ± 0.13 ^f^	1.67 ± 0.12 ^d^	6.56 ± 0.48 ^h^	0.99 ± 0.07 ^d^	1.22 ± 0.09 ^e^	0.52 ± 0.04 ^c^	14.62 ± 0.94 ^k^
Boreal Beast	3.13 ± 0.11 ^e^	1.11 ± 0.04 ^ab^	7.63 ± 0.28 ^k^	0.75 ± 0.03 ^c^	0.71 ± 0.03 ^d^	0.17 ± 0.01 ^b^	13.50 ± 0.49 ^i^
Boreal Blizzard	11.99 ± 0.43 ^k^	2.86 ± 0.10 ^h^	0.37 ± 0.01 ^b^	<LLQ	1.43 ± 0.05 ^f^	0.15 ± 0.01 ^b^	16.80 ± 0.61 ^l^
Aurora	4.38 ± 0.16 ^i^	1.46 ± 0.05 ^c^	5.15 ± 0.19 ^e^	0.36 ± 0.01 ^b^	2.32 ± 0.08 ^g^	0.12 ± 0.00 ^a^	13.78 ± 0.50 ^j^
Honeybee	3.76 ± 0.26 ^f^	6.66 ± 0.45 ^j^	7.47 ± 0.51 ^k^	0.65 ± 0.04 ^c^	0.21 ± 0.01 ^ab^	0.23 ± 0.02 ^b^	18.98 ± 1.30 ^n^
Vostorg	4.05 ± 0.15 ^h^	0.98 ± 0.004 ^a^	4.27 ± 0.15 ^c^	0.40 ± 0.01 ^b^	0.66 ± 0.01 ^d^	0.12 ± 0.00 ^a^	10.48 ± 0.38 ^e^
Jugana	15.46 ± 0.56 ^l^	9.08 ± 0.33 ^l^	0.40 ± 0.01 ^b^	0.07 ± 0.00 ^a^	3.13 ± 0.11 ^h^	0.17 ± 0.01 ^b^	28.31 ± 1.02 ^p^
Usłada	2.00 ± 0.07 ^c^	1.92 ± 0.07 ^e^	4.17 ± 0.15 ^c^	0.44 ± 0.02 ^b^	0.21 ± 0.01 ^ab^	0.14 ± 0.01 ^a^	8.89 ± 0.32 ^b^
Lawina	2.21 ± 0.08 ^c^	1.98 ± 0.07 ^e^	6.69 ± 0.24 ^hi^	0.74 ± 0.03 ^c^	0.51 ± 0.02 ^c^	0.09 ± 0.00 ^a^	12.22 ± 0.43 ^h^
Sinij Uties	2.52 ± 0.09 ^d^	1.79 ± 0.03 ^d^	6.87 ± 0.07 ^i^	0.72 ± 0.01 ^c^	0.49 ± 0.01 ^c^	0.09 ± 0.00 ^a^	12.47 ± 0.20 ^h^
Flesh							
Boreal Beauty	2.14 ± 0.10 ^c^	1.06 ± 0.05 ^ab^	5.71 ± 0.28 ^f^	0.67 ± 0.03 ^c^	1.17 ± 0.06 ^e^	0.45 ± 0.02 ^c^	11.20 ± 0.55 ^f^
Boreal Beast	0.96 ± 0.07 ^a^	1.02 ± 0.07 ^a^	7.90 ± 0.56 ^l^	1.09 ± 0.08 ^de^	0.57 ± 0.02 ^c^	0.18 ± 0.01 ^b^	11.73 ± 0.81 ^g^
Boreal Blizzard	2.97 ± 0.12 ^e^	2.72 ± 0.09 ^g^	0.21 ± 0.01 ^a^	0.52 ± 0.03 ^b^	0.67 ± 0.01 ^d^	0.18 ± 0.01 ^b^	7.27 ± 0.25 ^a^
Aurora	1.61 ± 0.10 ^b^	1.24 ± 0.04 ^b^	4.48 ± 0.34 ^c^	2.08 ± 0.16 ^g^	0.08 ± 0.02 ^a^	0.12 ± 0.01 ^a^	9.63 ± 0.72 ^d^
Honeybee	3.87 ± 0.38 ^g^	5.82 ± 0.41 ^i^	8.78 ± 0.37 ^m^	1.18 ± 0.11 ^e^	0.14 ± 0.02 ^a^	0.15 ± 0.01 ^b^	19.94 ± 1.31 ^o^
Vostorg	1.14 ± 0.04 ^a^	0.94 ± 0.03 ^a^	6.43 ± 0.22 ^g^	1.07 ± 0.04 ^de^	0.28 ± 0.01 ^b^	0.14 ± 0.00 ^a^	10.01 ± 0.34 ^e^
Jugana	5.33 ± 0.25 ^j^	8.44 ± 0.48 ^k^	0.46 ± 0.06 ^b^	3.07 ± 0.20 ^h^	0.21 ± 0.02 ^ab^	0.15 ± 0.01 ^b^	17.66 ± 1.26 ^m^
Usłada	1.16 ± 0.04 ^a^	2.11 ± 0.07 ^f^	4.90 ± 0.17 ^d^	0.89 ± 0.03 ^d^	0.07 ± 0.00 ^a^	0.12 ± 0.00 ^a^	9.25 ± 0.32 ^c^
Lawina	1.79 ± 0.06 ^b^	1.98 ± 0.07 ^e^	6.91 ± 0.22 ^i^	1.26 ± 0.02 ^f^	0.07 ± 0.00 ^a^	0.12 ± 0.00 ^a^	12.14 ± 0.41 ^h^
Sinij Uties	1.24 ± 0.06 ^a^	1.28 ± 0.06 ^b^	7.09 ± 0.45 ^j^	1.32 ± 0.10 ^f^	0.09 ± 0.00 ^a^	0.10 ± 0.00 ^a^	11.12 ± 0.54 ^f^
Mean							
Skin	5.32	2.95	4.96	0.51	1.09	0.18	15.01
Flesh	2.22	2.66	5.29	1.32	0.34	0.17	11.99

Values are mean (*n* = 3) and SD. Values followed by different letters (a–p) in the same column indicate significant differences (*p* < 0.05). Legend: <LLQ, value below detection limit.

**Table 4:** Content of organic acids and sugars in the fruit skin and flesh of 10 cultivars of haskap berries.

Cultivars and Parts	Organic Acids (g/100 g d.m.)				Sugars (g/100 g d.m.)		
	Citric Acid	Malic Acid	Quinic Acid	Total Acids	Glucose	Fructose	Total Sugars
Skin							
Boreal Beauty	0.90 ± 0.07 ^a^	nd	0.46 ± 0.04 ^a^	1.37 ± 0.31 ^b^	0.44 ± 0.02 ^a^	0.54 ± 0.01 ^a^	0.98 ± 0.06 ^a^
Boreal Beast	0.35 ± 0.03 ^a^	nd	0.13 ± 0.01 ^a^	0.47 ± 0.16 ^a^	2.20 ± 0.08 ^c^	2.62 ± 1.00 ^bcd^	4.82 ± 0.24 ^d^
Boreal Blizzard	0.86 ± 0.07 ^a^	nd	0.35 ± 0.03 ^a^	1.21 ± 0.36 ^b^	2.05 ± 0.07 ^bc^	2.33 ± 0.11 ^ab^	4.38 ± 0.17 ^d^
Aurora	0.95 ± 0.06 ^a^	nd	0.33 ± 0.02 ^a^	1.28 ± 0.13 ^b^	2.24 ± 0.08 ^c^	2.72 ± 0.07 ^bcd^	5.01 ± 0.28 ^e^
Honeybee	1.06 ± 0.08 ^a^	nd	0.32 ± 0.03 ^a^	1.37 ± 0.52 ^b^	1.62 ± 0.06 ^bc^	2.32 ± 0.10 ^b^	3.94 ± 0.40 ^c^
Vostorg	1.25 ± 0.09 ^a^	nd	0.50 ± 0.02 ^a^	1.75 ± 0.53 ^c^	0.99 ± 0.02 ^ab^	1.19 ± 0.03 ^a^	2.18 ± 0.12 ^b^
Jugana	1.01 ± 0.105 ^a^	nd	0.35 ± 0.02 ^a^	1.35 ± 0.47 ^b^	2.04 ± 0.12 ^bc^	2.35 ± 0.07 ^ab^	4.39 ± 0.18 ^d^
Usłada	0.58 ± 0.00 ^a^	nd	0.17 ± 0.01 ^a^	0.75 ± 0.29 ^a^	3.53 ± 0.12 ^d^	3.65 ± 0.13 ^de^	7.18 ± 0.07 ^f^
Lawina	0.59 ± 0.05 ^a^	nd	0.22 ± 0.01 ^a^	0.81 ± 0.27 ^a^	2.28 ± 0.08 ^c^	2.32 ± 0.08 ^b^	4.60 ± 0.03 ^d^
Sinij Uties	0.56 ± 0.06 ^a^	nd	0.43 ± 0.01 ^a^	0.98 ± 0.09 ^ab^	3.90 ± 0.14 ^d^	3.99 ± 0.14 ^e^	7.89 ± 0.06 ^g^
Flesh							
Boreal Beauty	19.51 ± 1.52 ^h^	2.97 ± 0.23 ^d^	5.45 ± 0.42 ^e^	27.93 ± 8.92 ^i^	19.32 ± 0.68 ^e^	22.54 ± 0.79 ^g^	41.86 ± 2.27 ^i^
Boreal Beast	7.63 ± 0.59 ^b^	1.86 ± 0.14 ^c^	2.45 ± 0.19 ^c^	11.94 ± 3.17 ^d^	18.85 ± 0.66 ^e^	20.14 ± 0.71 ^f^	38.99 ± 0.91 ^h^
Boreal Blizzard	9.67 ± 0.75 ^c^	1.77 ± 0.14 ^c^	1.47 ± 0.11 ^b^	12.91 ± 4.65 ^e^	22.77 ± 0.80 ^f^	24.66 ± 0.86 ^h^	47.43 ± 1.34 ^j^
Aurora	10.92 ± 0.82 ^d^	1.73 ± 0.13 ^c^	2.30 ± 0.18 ^c^	14.94 ± 5.15 ^f^	19.12 ± 0.67 ^e^	21.60 ± 0.77 ^g^	40.72 ± 1.75 ^h^
Honeybee	12.14 ± 0.94 ^e^	1.33 ± 0.10 ^b^	5.69 ± 0.44 ^e^	19.16 ± 5.44 ^h^	18.70 ± 0.65 ^e^	22.07 ± 0.76 ^g^	40.81 ± 2.39 ^h^
Vostorg	15.06 ± 1.17 ^g^	16.08 ± 0.47 ^f^	17.37 ± 0.98 ^f^	48.51 ± 1.15 ^j^	27.67 ± 0.90 ^i^	29.43 ± 1.03 ^j^	57.10 ± 1.24 ^n^
Jugana	14.53 ± 1.13 ^f^	1.03 ± 0.08 ^a^	2.16± 0.17 ^c^	17.72 ± 7.49 ^g^	25.22 ± 0.88 ^h^	27.45 ± 0.94 ^i^	52.66 ± 1.58 ^l^
Usłada	9.57 ± 0.74 ^c^	3.47 ± 0.27 ^e^	2.41 ± 0.19 ^c^	15.45 ± 3.86 ^f^	30.91 ± 1.08 ^j^	33.82 ± 1.18 ^k^	64.73 ± 2.05 ^o^
Lawina	9.54 ± 0.73 ^c^	2.03 ± 0.15 ^c^	3.35 ± 0.26 ^d^	14.86 ± 4.03 ^f^	24.18 ± 0.85 ^g^	24.64 ± 0.86 ^h^	48.82 ± 0.33 ^k^
Sinij Uties	8.82 ± 0.72 ^c^	1.80 ± 0.14 ^c^	2.61 ± 0.20 ^c^	13.24 ± 3.84 ^e^	27.08 ± 1.01 ^i^	28.47 ± 1.00 ^ij^	55.56 ± 0.98 ^m^
Mean							
Skin	0.81	0.00	0.32	1.13	2.13	2.40	4.53
Flesh	11.74	3.40	4.53	19.67	23.38	25.48	48.94

Values are mean (*n* = 3) and SD. Values followed by different letters (a–o) in the same column indicate significant differences (*p* < 0.05). Legend: nd, not identified.

**Table 5:** Antioxidant (ABTS, CUPRAC, ChP, OH˙ and O_2_˙^−^), antidiabetic (α-amylase, α-glucosidase) and anti-inflammatory (LOXI, XOI) activity in the fruit skin and flesh of 10 haskap berry cultivars.

Cultivars and Parts			Antioxidant			Antidiabetic		Anti- Inflammatory	
	ABTS (mmol TE/100 g)	CUPRAC (mmol TE/100 g)	ChP (μg/mL)	OH˙ (μg/mL)	O_2_˙^−^ (μg/mL)	α-Amylase (mg/mL)	α-Glucosidase (mg/mL)	LOXI (mg/mL)	XOI (mg/mL)
Skin									
Boreal Beauty	68.64 ± 1.80 ^g^	48.03 ± 0.61 ^k^	428.91 ± 3.01 ^e^	800.41 ± 12.00 ^j^	687.34 ± 18.38 ^g^	11.92 ± 0.18 ^e^	10.14 ± 1.07 ^ab^	0.35 ± 0.02 ^a^	1.93 ± 0.08 ^de^
Boreal Beast	98.01 ± 3.61 ^l^	54.83 ± 0.09 ^n^	259.09 ± 4.77 ^a^	408.11 ± 3.86 ^c^	423.14 ± 12.61 ^b^	6.19 ± 0.11 ^ab^	15.26 ± 1.02 ^cde^	0.30 ± 0.03 ^a^	1.83 ± 0.19 ^cd^
Boreal Blizzard	84.42 ± 2.31 ^k^	48.82 ± 0.11 ^l^	315.21 ± 5.75 ^b^	499.50 ± 13.03 ^e^	516.82 ± 5.51 ^d^	7.89 ± 0.57 ^bc^	11.97 ± 0.31 ^abcd^	0.61 ± 0.04 ^cd^	2.12 ± 0.15 ^e^
Aurora	74.70 ± 1.77 ^h^	41.32 ± 0.60 ^i^	373.51 ± 11.11 ^d^	576.66 ± 4.85 ^g^	765.72 ± 7.65 ^h^	8.49 ± 0.48 ^cd^	13.16 ± 1.01 ^bcd^	0.79 ± 0.03 ^e^	1.53 ± 0.12 ^b^
Honeybee	103.65 ± 2.16 ^m^	63.91 ± 0.38 ^o^	240.04 ± 4.42 ^a^	334.15 ± 5.07 ^a^	371.70 ± 11.76 ^a^	4.84 ± 0.33 ^a^	8.35 ± 0.33 ^a^	0.46 ± 0.04 ^b^	1.11 ± 0.05 ^a^
Vostorg	79.94 ± 1.71 ^i^	49.78 ± 0.65 ^m^	366.37 ± 7.80 ^d^	718.86 ± 5.16 ^i^	533.54 ± 3.77 ^d^	5.24 ± 0.24 ^a^	17.80 ± 0.80 ^e^	0.57 ± 0.05 ^c^	1.54 ± 0.13 ^b^
Jugana	75.86 ± 1.42 ^h^	42.23 ± 0.38 ^j^	381.61 ± 2.84 ^d^	833.61 ± 4.28 ^l^	602.42 ± 3.10 ^e^	6.92 ± 0.40 ^abc^	11.33 ± 0.56 ^abc^	0.60 ± 0.02 ^cd^	1.83 ± 0.15 ^cd^
Usłada	83.54 ± 1.34 ^jk^	48.77 ± 0.33 ^l^	346.22 ± 11.11 ^c^	368.04 ± 3.02 ^b^	464.76 ± 9.91 ^c^	4.86 ± 0.44 ^a^	10.97 ± 0.42 ^abc^	0.69 ± 0.06 ^d^	1.73 ± 0.16 ^bcd^
Lawina	83.91 ± 1.45 ^jk^	49.31 ± 0.21 ^m^	335.61 ± 9.90 ^c^	549.51 ± 6.72 ^f^	652.51 ± 5.02 ^f^	6.41 ± 0.82 ^ab^	14.57 ± 1.34 ^bcde^	0.76 ± 0.05 ^e^	1.90 ± 0.21 ^de^
Sinij Uties	81.33 ± 2.51 ^ij^	54.69 ± 0.90 ^n^	330.22 ± 9.04 ^bc^	443.43 ± 4.21 ^d^	454.56 ± 9.62 ^c^	5.19 ± 0.31 ^a^	16.04 ± 0.72 ^de^	0.65 ± 0.05 ^cd^	1.74 ± 0.07 ^bcd^
Flesh									
Boreal Beauty	39.88 ± 0.82 ^de^	24.52 ± 0.25 ^f^	828.11 ± 7.37 ^j^	1268.05 ± 7.21 ^o^	1108.23 ± 12.42 ^k^	21.94 ± 1.08 ^i^	25.62 ± 2.86 ^f^	1.36 ± 0.11 ^h^	2.89 ± 0.12 ^i^
Boreal Beast	36.70 ± 0.74 ^bc^	21.88 ± 0.47 ^c^	890.66 ± 6.27 ^k^	951.12 ± 10.35 ^m^	764.91 ± 2.86 ^h^	17.03 ± 1.57 ^fg^	33.04 ± 2.18 ^g^	1.20 ± 0.07 ^g^	2.84 ± 0.12 ^ghi^
Boreal Blizzard	40.83 ± 0.83 ^e^	23.94 ± 1.02 ^e^	806.02 ± 5.31 ^i^	826.07 ± 9.55 ^kl^	845.42 ± 8.88 ^j^	15.01 ± 1.23 ^f^	37.47 ± 3.45 ^h^	1.14 ± 0.05 ^g^	3.06 ± 0.22 ^i^
Aurora	42.37± 0.90 ^e^	24.64 ± 0.85 ^f^	537.23 ± 15.90 ^g^	809.22 ± 5.28 ^jk^	1217.32 ± 3.28 ^l^	17.0 1± 0.92 ^fg^	32.42 ± 2.38 ^g^	1.17 ± 0.05 ^g^	1.59 ± 0.12 ^bc^
Honeybee	48.72 ± 0.81 ^f^	30.17 ± 0.32 ^h^	439.20 ± 10.60 ^e^	680.21 ± 9.01 ^h^	608.01 ± 9.32 ^e^	10.12 ± 1.62 ^de^	26.10 ± 4.62 ^f^	0.98 ± 0.04 ^f^	2.42 ± 0.20 ^f^
Vostorg	34.41 ± 0.72 ^b^	20.34 ± 0.11 ^a^	1241.30 ± 26.41 ^m^	1414.23 ± 11.42 ^r^	856.82 ± 5.01 ^j^	15.54 ± 2.30 ^fg^	43.86 ± 2.04 ^i^	1.12 ± 0.07 ^g^	2.61 ± 0.04 ^fg^
Jugana	37.72 ± 0.65 ^cd^	21.47 ± 0.50 ^b^	906.63 ± 6.42 ^k^	993.94 ± 6.30 ^n^	820.37 ± 10.42 ^i^	17.29 ± 0.58 ^gh^	27.93 ± 1.52 ^f^	1.15 ± 0.09 ^g^	2.74 ± 0.11 ^gh^
Usłada	29.40 ± 0.36 ^a^	20.34 ± 0.77 ^a^	1122.41 ± 24.22 ^l^	802.77 ± 4.33 ^j^	860.32 ± 5.60 ^j^	19.13 ± 1.62 ^h^	44.48 ± 3.50 ^i^	1.10 ± 0.02 ^g^	3.77 ± 0.07 ^k^
Lawina	41.86 ± 0.37 ^e^	25.35 ± 0.27 ^g^	730.42 ± 9.40 ^h^	1361.09 ± 6.60 ^p^	1627.62 ± 15.72 ^m^	16.23 ± 2.10 ^fg^	34.54 ± 2.02 ^gh^	1.14 ± 0.05 ^g^	3.50 ± 0.16 ^j^
Sinij Uties	40.52 ± 0.71 ^e^	23.68 ± 1.01 ^d^	501.67 ± 13.66 ^f^	725.31 ± 6.89 ^i^	743.51 ± 5.42 ^h^	15.68 ± 1.55 ^fg^	46.54 ± 1.90 ^i^	1.14 ± 0.05 ^g^	3.57 ± 0.15 ^jk^
Mean									
Skin	83.40	50.21	338.27	553.20	547.31	6.79	12.96	0.58	1.73
Flesh	39.22	23.58	799.38	983.24	945.23	16.50	35.20	1.15	2.90

Values are mean (*n* = 3) and SD. Values followed by different letters (a–r) in the same column indicate significant differences (*p* < 0.05).

**Table 6:** Cytotoxic activity of the fruit skin and flesh of 10 haskap berry cultivars against six cancer cell lines.

Cultivars and Parts	IC_50_ value (μg/mL)					
	Caco-2	Ht-29	U251 mg	U87 mg	AGS	SK-Mel-29
Skin						
Boreal Beauty	256.21 ± 5.41 ^h^	233.11 ± 3.33 ^f^	374.11 ± 2.76 ^b^	425.28 ± 3.15 ^h^	283.71 ± 6.02 ^g^	415.35 ± 12.10 ^b^
Boreal Beast	122.22 ± 6.02 ^b^	218.07 ± 3.11 ^de^	432.88 ± 5.56 ^c^	289.56 ± 1.65 ^b^	182.11 ± 5.45 ^cd^	494.76 ± 7.02 ^cd^
Boreal Blizzard	175.67 ± 3.66 ^de^	390.18 ± 6.10 ^k^	493.45 ± 5.00 ^d^	364.85 ± 9.81 ^e^	187.43 ± 1.51 ^d^	485.37 ± 6.88 ^c^
Aurora	287.55 ± 13.02 ^i^	308.64 ± 4.41 ^hi^	630.89 ± 10.21 ^h^	419.73 ± 3.13 ^gh^	208.85 ± 8.11 ^e^	584.61 ± 8.26 ^g^
Honeybee	107.30 ± 4.65 ^a^	96.04 ± 3.78 ^a^	351.10 ± 8.00 ^a^	249.78 ± 5.66 ^a^	155.03 ± 3.57 ^a^	333.10 ± 4.65 ^a^
Vostorg	188.10 ± 9.28 ^f^	176.91 ± 5.47 ^c^	588.54 ± 9.31 ^g^	411.91 ± 4.01 ^g^	240.00 ± 11.88 ^f^	584.62 ± 8.28 ^g^
Jugana	206.08 ± 4.43 ^g^	226.28 ± 3.21 ^ef^	554.41 ± 10.82 ^f^	390.67 ± 8.33 ^f^	236.88 ± 5.02 ^f^	587.11 ± 11.85 ^g^
Usłada	140.91 ± 6.21 ^c^	131.45 ± 7.07 ^b^	352.52 ± 8.86 ^a^	312.65 ± 1.89 ^c^	164.06 ± 4.16 ^ab^	426.91 ± 8.58 ^b^
Lawina	165.88 ± 3.51 ^d^	301.43 ± 4.31 ^h^	539.12 ± 12.18 ^f^	310.40 ± 1.71 ^c^	187.37 ± 2.02 ^d^	584.62 ± 8.28 ^g^
Sinij Uties	182.56 ± 2.85 ^ef^	121.31 ± 8.40 ^b^	478.03 ± 4.77 ^d^	307.71 ± 8.00 ^c^	172.77 ± 0.88 ^bc^	506.04 ± 7.18 ^de^
Flesh						
Boreal Beauty	399.30 ± 10.35 ^l^	336.71 ± 6.01 ^j^	518.45 ± 3.89 ^e^	571.54 ± 9.61 ^l^	387.41 ± 3.41 ^k^	550.81 ± 9.81 ^f^
Boreal Beast	323.33 ± 2.55 ^j^	315.10 ± 5.62 ^i^	624.61 ± 6.02 ^h^	356.88 ± 7.89 ^e^	353.11 ± 6.12 ^i^	714.72 ± 12.65 ^h^
Boreal Blizzard	416.38 ± 3.60 ^m^	538.33 ± 9.57 ^m^	>750	500.91 ± 7.71 ^k^	383.40 ± 6.52 ^k^	481.22 ± 8.66 ^c^
Aurora	693.08 ± 6.03 ^r^	445.72 ± 7.88 ^l^	>750	564.90 ± 4.22 ^l^	371.30 ± 3.58 ^j^	>750
Honeybee	294.61 ± 3.11 ^i^	208.22 ± 9.35 ^d^	515.78 ± 13.01 ^e^	342.67 ± 3.78 ^d^	304.73 ± 7.03 ^h^	701.21 ± 12.51 ^h^
Vostorg	458.24 ± 4.22 ^o^	257.68 ± 4.57 ^g^	>750	603.03 ± 2.54 ^m^	418.32 ± 4.03 ^l^	>750
Jugana	488.54 ± 4.22 ^p^	327.03 ± 5.77 ^j^	>750	568.57 ± 8.72 ^l^	367.28 ± 7.78 ^j^	>750
Usłada	350.72 ± 12.81 ^k^	264.41 ± 12.78 ^g^	513.16 ± 8.04 ^e^	481.16 ± 3.10 ^j^	235.09 ± 7.16 ^f^	522.80 ± 9.27 ^e^
Lawina	393.11 ± 3.40 ^l^	435.38 ± 7.68 ^l^	>750	477.38 ± 7.26 ^j^	384.00 ± 9.38 ^k^	>750
Sinij Uties	432.60 ± 3.68 ^n^	209.88 ± 3.18 ^d^	739.02 ± 7.37 ^i^	440.73 ± 0.94 ^i^	301.72 ± 7.88 ^h^	>750
Mean						
Skin	183.33	220.32	479.45	348.11	201.78	500.21
Flesh	425.04	333.78	717.91	491.26	350.60	672.13

Values are mean (*n* = 9) and SD. Values followed by different letters (a–r) in the same column indicate significant differences (*p* < 0.05).

## Data Availability

Data are available from the authors.

## Supplementary Materials

The following supporting information can be downloaded at: https://www.mdpi.com/article/10.3390/ijms26146618/s1.
