# Supplementary material for: Bioactive Compounds and In Vitro Health-Promoting Activity of the Fruit Skin and Flesh of Different Haskap Berry (Lonicera caerulea var. kamtschatica Sevast.) Cultivars
Source: Int J Mol Sci. 2025 Jul 10;26(14):6618. doi: 10.3390/ijms26146618 (PMC12294290; doi:10.3390/ijms26146618)
Supplement: Supplementary file 1 [file ijms-26-06618-s001.zip › ijms-3728269-supplementary.pdf]

Supplementary material

Bioactive compounds and in vitro health-promoting activity of the fruit skin and flesh of different haskap berry (*Lonicera caerulea* var. *kamtschatica* Sevast.) cultivars

Natalia Żurek[1,*], Stanisław Pluta[2], Michał Świeca[3], Leszek Potocki[4], Łukasz Seliga[2], Ireneusz Kapusta[1]

1 Department of Food Technology and Human Nutrition, University of Rzeszow, 4 Zelwerowicza St., 35-601 Rzeszow, Poland
2 Department of Horticultural Crop Breeding, National Institute of Horticultural Research (InHort), Konstytucji 3 Maja 1/3 St., 96-100 Skierniewice, Poland
3 Department of Food Chemistry and Biochemistry, University of Life Sciences in Lublin, 8 Skromna St., 20-704 Lublin, Poland
4 Department of Biotechnology, University of Rzeszow, 1 Pigonia St., 35-310 Rzeszow, Poland
* Correspondence: nzurek@ur.edu.pl; Tel.: +48-17-785-5236

Content:

Table S1. Individual polyphenolic compounds and iridoids identified in the skin and flesh of ten haskap berry varieties by UPLC-PDA-MS/MS.

Table S2. Positive controls in assays for antioxidant, antidiabetic, anti-inflammatory and anticancer activity.

Table S3. Correlation between the analyzed parameters for the skin of haskap fruit.

Table S4. Correlation between the analyzed parameters for the haskap fruit flesh.

Figure S1. A. Ethanolic *Lonicera caerulea* berries extracts (1-10) mediated changes in the growth of three strains of Gram-negative bacteria (*E. coli* PCM 2209, *S. enterica* sv. *Enteritidis*, *P. aeruginosa* DSM 19880), three strains of Gram-positive bacteria (*S. aureus* PCM 458, *L. monocytogenes* PCM 2191, and *E. faecalis*), and one strain of yeast (*C. albicans* ATCC 14053). B. Table 1. Minimum Inhibitory Concentration (MIC) of ethanolic skin and flesh *Lonicera caerulea* berry extracts.

Table S1. Individual polyphenolic compounds and iridoids identified in the skin and flesh of ten haskap berry varieties by UPLC-PDA-MS/MS.

No.	Rt	λ_{max}	[M-H] m/z		Compound
	min	nm	MS	MS/MS	
**Anthocyanins**					
1	2.02	279, 520	737$^+$	575, 287	(Epi)catechin-Cyanidin 3-*O*-glucoside
2	2.11	279, 524	773$^+$	611, 287	Cyanidin 3-*O*-sophoroside-5-*O*-glucoside
3	2.18	281, 519	757$^+$	595, 287	Cyanidin 3-*O*-rutinoside-5-*O*-glucoside
4	2.23	279, 515	611$^+$	449, 287	Cyanidin 3,5-*O*-diglucoside
5	2.66	279, 514	449$^+$	287	Cyanidin 3-*O*-glucoside
6	2.85	279, 515	595$^+$	287	Cyanidin 3-*O*-rutinoside
7	3.09	278, 504	433$^+$	271	Pelargonidin 3-*O*-glucoside
8	3.35	279, 517	463$^+$	301	Peonidin 3-*O*-glucoside
9	3.43	279, 517	609$^+$	301	Peonidin 3-*O*-rutinoside
10	3.74	277, 531	449$^+$	303	Delphinidin 3-*O*-rhamnoside
**Other phenolics**					
11	2.34	288sh, 324	353-	191	Neochlorogenic acid
12	2.53	279	577-	289	Procyanidin dimer B-type
13	2.93	288sh, 324	353-	191	Chlorogenic acid
14	3.07	279	577-	289	Procyanidin dimer B-type
15	3.13	281, 339	447-	285	Luteolin 7-*O*-glucoside
16	3.95	255, 354	755-	301	Quercetin 3-*O*-rutinoside-7-*O*-rhamnoside
17	4.13	255, 355	595-	301	Quercetin 3-*O*-pentoside-glucoside I
18	4.43	255, 355	609-	301	Quercetin 3-*O*-rutinoside
19	4.63	255, 355	463-	301	Quercetin 3-*O*-glucoside
20	4.76	255, 355	447-	301	Quercetin 3-*O*-rhmanoside
21	4.80	264, 336	593-	447, 285	Kaempferol 3-*O*-glucoside-7-*O*-rhamnoside
22	4.94	264, 338	593-	285	Kaempferol 3-*O*-rutinoside
23	5.09	264, 339	623	447, 285	Kaempferol 3-O-glucoside-7-*O*-glucuronide
24	5.13	288sh, 324	515-	353	3,4-di-*O*-caffeoyl-quinic acid
25	5.35	255, 335	505-	301	Quercetin 3-*O*-(6”-acetylo)-glucoside
26	5.47	264, 338	431-	285	Kaempferol 3-*O*-rhamnoside
27	6.37	264, 338	417-	285	Kaempferol 3-*O*-pentoside I
28	6.71	264, 336	417-	285	Kaempferol 3-*O*-pentoside II
29	7.18	255, 350	447	301	Quercetin 3-*O*-rhamnoside
**Iridoids**					
30	2.66		375 [M-H]$^-$<br>751 [2M-H]$^-$	389, 227	Loganic acid
31	3.70		489 [M-H]$^-$<br>535 [M-H+46]$^-$	195, 125	Sweroside pentoside isomer I
32	3.74		389 [M-H]$^-$<br>435 [M-H+46]$^-$	227	Loganin
33	3.78		521 [M-H]$^-$<br>567 [M-H+46]$^-$	389, 227	Loganin pentoside
34	3.81		357 [M-H]$^-$<br>403 [M-H+46]$^-$	125	Sweroside

35	4.07	489 [M-H]$^-$ 535 [M-H+46]$^-$	195, 125	Sweroside pentoside isomer II

Abbreviations: Rt, retention time; [M-H]$^-$, negative ion values; m/z, mass-to-charge ratio.

Table S2. Positive controls in assays for antioxidant, antidiabetic, anti-inflammatory and anticancer activity.

	ABTS (mmol TE/g)	CUPRAC (mmol TE/g)	ChP (µg/ml)	$OH^{\cdot}$ (µg/ml)	$O_2^{\cdot -}$ (µg/ml)	α-amylase (mg/ml)	α-glucosidase (mg/ml)	LOXI (mg/ml)	XOI (mg/ml)	Caco-2 (µg/ml)	Ht-29 (µg/ml)	U251mg (µg/ml)	U87mg (µg/ml)	AGS (µg/ml)	SK-Mel-29 (µg/ml)
Ascorbic acid	56.9 ± 0.0	66.2 ± 0.1			75.8 ± 0.0										
EDTA			12.9 ± 0.04												
Quercetin				9.9 ± 0.0											
Acarbose						0.05 ± 0.0	0.2 ± 0.0								
Allopurynol									0.008975 ± 0.0						
Quercetin								0.0895 ± 0.0	0.0127 ± 0.0						
Cisplatine										1.5 ± 0.0	11.2 ± 0.3	24.2 ± 0.8	12.7 ± 0.2	6.3 ± 0.2	25.8 ± 0.6

Table S3. Correlation between the analyzed parameters for the skin of haskap fruit.

Ash	SM	SSC	TA	Organic acids	Sugars	ABTS	CUPRAC	ChP	O2	OH	α-amylase	α-glucosidase	XOI	LOX	HT-29	SK-Mel-29	Caco-2	AGS	U87mg	U251mg	Anthocyanins	Other phenolics	Total PC	Iridoids	
1.00	-0.16	-0.08	0.07	0.02	-0.27	-0.08	-0.12	0.14	-0.03	0.10	0.11	0.20	0.22	0.39	-0.03	0.23	-0.32	-0.08	-0.15	0.01	0.35	0.60	0.41	0.41	**Ash**
	1.00	-0.04	0.52	0.19	-0.11	0.31	0.35	-0.31	-0.30	-0.12	-0.09	-0.21	-0.65	0.10	-0.25	-0.17	-0.15	-0.23	-0.10	0.13	-0.05	-0.20	0.09	-0.08	**SM**
		1.00	-0.17	0.26	0.60	0.23	0.11	-0.27	-0.13	-0.69	-0.61	-0.68	-0.30	0.40	-0.26	-0.27	-0.14	-0.68	-0.47	-0.27	-0.29	-0.20	0.59	-0.27	**SSC**
			1.00	0.47	-0.19	-0.36	-0.06	0.44	0.29	0.49	-0.54	-0.58	-0.51	0.16	-0.37	0.05	0.38	0.41	0.34	0.20	-0.07	0.01	0.19	-0.05	**TA**
				1.00	0.66	-0.60	-0.54	0.52	0.61	0.59	-0.35	-0.52	0.66	0.74	0.48	0.45	0.60	0.61	0.60	0.44	-0.68	-0.88	-0.91	-0.77	**Organic acids**
					1.00	-0.84	-0.86	0.88	0.76	0.68	0.86	0.72	0.62	0.73	0.61	0.40	0.66	0.66	0.56	0.59	0.87	0.90	0.92	0.71	**Sugars**
						1.00	0.81	-0.96	-0.78	-0.76	-0.73	-0.56	-0.38	-0.29	-0.33	-0.45	-0.87	-0.74	-0.85	-0.42	0.96	0.79	0.95	0.37	**ABTS**
							1.00	-0.78	-0.81	-0.66	-0.71	-0.48	-0.45	-0.45	-0.58	-0.68	-0.76	-0.55	-0.80	-0.62	0.97	0.66	0.94	0.43	**CUPRAC**
								1.00	0.76	0.81	0.78	0.79	0.32	0.22	0.25	0.40	0.81	0.79	0.85	0.33	-0.83	-0.59	-0.82	-0.35	**ChP**
									1.00	0.67	0.82	0.77	0.28	0.39	0.60	0.54	0.89	0.65	0.77	0.57	-0.97	-0.84	-0.96	-0.20	**O2**
										1.00	0.65	0.61	0.28	-0.04	0.30	0.50	0.68	0.93	0.83	0.46	-0.85	-0.80	-0.83	-0.44	**OH**
											1.00	0.89	0.33	0.21	0.09	0.29	0.36	0.25	0.29	0.21	-0.91	-0.87	-0.88	-0.33	**α-amylase**
												1.00	0.45	0.36	0.19	0.22	0.32	0.28	0.33	0.25	-0.81	0.70	-0.78	-0.38	**α-glucosidase**
													1.00	0.10	0.59	0.27	0.19	0.26	0.28	0.09	-0.82	-0.76	-0.80	-0.25	**XOI**
														1.00	0.25	0.50	0.26	0.25	0.12	0.53	-0.93	-0.90	-0.91	-0.13	**LOX**
															1.00	0.46	0.44	0.25	0.46	0.50	-0.88	-0.77	-0.81	-0.31	**Ht-29**
																1.00	0.44	0.29	0.49	0.92	-0.54	-0.43	-0.50	-0.51	**Sk-Mel-29**
																	1.00	0.70	0.87	0.54	-0.87	-0.78	-0.83	-0.40	**Caco-2**
																		1.00	0.85	0.27	-0.81	-0.77	-0.72	-0.10	**AGS**
																			1.00	0.55	-0.62	-0.51	-0.66	-0.48	**U87mg**
																				1.00	-0.56	-0.56	-0.59	-0.31	**U251mg**
																					1.00	0.90	0.98	0.89	**Anthocyanins**
																						1.00	0.99	0.94	**Other phenolics**
																							1.00	0.90	**Total PC**
																								1.00	**Iridoids**

Table S4. Correlation between the analyzed parameters for the haskap fruit flesh.

Ash	SM	SSC	TA	Organic acids	Sugars	ABTS	CUPRAC	ChP	O2	OH	α-amylase	α-glucosidase	XOI	LOX	HT-29	SK-Mel-29	Caco-2	AGS	U87mg	U251mg	Anthocyanins	Other phenolics	Total PC	Iridoids	
1.00	0.08	0.30	0.16	-0.39	-0.25	-0.44	-0.36	0.52	0.22	0.33	0.11	0.26	0.39	-0.43	-0.08	0.31	-0.14	-0.13	0.13	0.20	0.14	0.47	0.44	0.31	**Ash**
	1.00	0.22	0.56	-0.17	-0.13	0.46	0.43	-0.28	-0.42	-0.32	-0.16	-0.08	-0.42	-0.68	-0.20	0.24	0.12	0.00	-0.06	0.23	-0.17	-0.10	-0.14	-0.17	**SM**
		1.00	0.47	-0.57	-0.60	0.37	0.50	-0.48	0.18	-0.12	-0.56	-0.59	0.17	-0.35	-0.43	0.33	-0.13	-0.42	-0.24	-0.31	0.09	0.32	0.19	0.17	**SSC**
			1.00	-0.08	-0.20	0.21	0.20	-0.03	0.03	0.28	-0.36	-0.16	-0.34	-0.18	-0.37	0.42	0.30	0.19	0.49	0.03	-0.11	0.11	-0.05	-0.07	**TA**
				1.00	0.73	-0.86	-0.85	0.75	0.63	0.68	-0.20	-0.13	0.71	0.81	0.44	0.51	0.64	0.75	0.73	0.49	-0.67	-0.83	-0.87	-0.66	**Organic acids**
					1.00	-0.93	-0.93	0.85	0.63	0.68	0.88	0.92	0.85	0.87	0.65	0.43	0.78	0.77	0.70	0.68	-0.91	-0.89	-0.90	0.67	**Sugars**
						1.00	0.93	-0.86	0.08	-0.24	-0.86	-0.74	-0.41	-0.16	0.13	0.17	0.04	0.20	-0.33	0.06	0.88	0.80	0.91	0.38	**ABTS**
							1.00	-0.82	0.06	-0.30	-0.71	-0.81	-0.28	-0.26	0.02	-0.01	-0.17	-0.04	-0.46	-0.22	0.80	0.71	0.85	0.20	**CUPRAC**
								1.00	-0.04	0.54	0.55	0.57	0.27	0.15	-0.04	-0.12	-0.14	0.15	0.44	0.05	-0.67	-0.63	-0.77	-0.24	**ChP**
									1.00	0.56	0.51	0.43	0.03	0.32	0.56	0.29	0.35	0.39	0.37	0.31	-0.47	-0.58	-0.64	-0.35	**O2**
										1.00	0.58	0.48	0.08	0.38	0.14	0.31	0.02	0.68	0.53	0.23	-0.79	-0.66	-0.93	-0.43	**OH**
											1.00	0.88	0.37	0.33	0.11	0.38	0.28	0.19	0.41	0.23	-0.82	-0.76	-0.80	-0.41	**α-amylase**
												1.00	0.41	0.28	0.15	0.30	0.36	0.21	0.34	0.23	-0.80	0.71	-0.77	-0.40	**α-glucosidase**
													1.00	0.09	-0.18	-0.41	-0.59	-0.43	-0.21	-0.20	-0.79	-0.68	-0.78	-0.22	**XOI**
														1.00	0.24	-0.20	0.16	0.34	0.38	-0.06	-0.73	-0.60	-0.77	-0.44	**LOX**
															1.00	-0.13	0.41	0.50	0.31	0.60	-0.61	-0.05	-0.58	-0.44	**Ht-29**
																1.00	0.46	0.37	0.17	0.43	-0.58	-0.22	-0.58	-0.12	**Sk-Mel-29**
																	1.00	0.37	0.66	0.60	-0.75	-0.10	-0.44	-0.41	**Caco-2**
																		1.00	0.52	0.61	-0.49	-0.03	-0.55	-0.10	**AGS**
																			1.00	0.46	-0.56	-0.27	-0.63	-0.32	**U87mg**
																				1.00	-0.65	-0.24	-0.62	-0.48	**U251mg**
																					1.00	0.67	0.90	0.94	**Anthocyanins**
																						1.00	0.62	0.84	**Other phenolics**
																							1.00	0.89	**Total PC**
																								1.00	**Iridoids**

A.

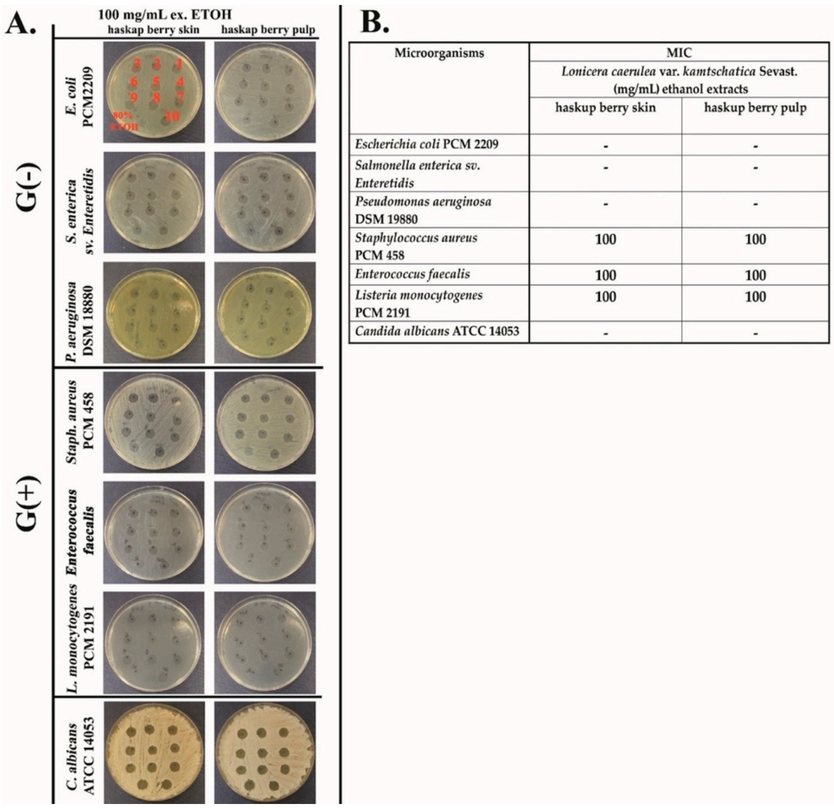

B.

Microorganisms	MIC	
	Lonicera caerulea var. *kamtschatica* Sevast. (mg/mL) ethanol extracts	
	haskup berry skin	haskup berry pulp
Escherichia coli PCM 2209	-	-
Salmonella enterica sv. Enteretidis	-	-
Pseudomonas aeruginosa DSM 19880	-	-
Staphylococcus aureus PCM 458	100	100
Enterococcus faecalis	100	100
Listeria monocytogenes PCM 2191	100	100
Candida albicans ATCC 14053	-	-

Figure S1. A. Ethanolic *Lonicera caerulea* berries extracts (1-10) mediated changes in the growth of three strains of Gram-negative bacteria (*Escherichia coli* PCM 2209, *Salmonella enterica* sv. *Enteritidis*, *Pseudomonas aeruginosa* DSM 19880), three strains of Gram-positive bacteria (*Staphylococcus aureus* PCM 458, *Listeria monocytogenes* PCM 2191, and *Enterococcus faecalis*), and one strain of yeast (*Candida albicans* ATCC 14053). **B**. Table 1. Minimum Inhibitory Concentration (MIC) of ethanolic skin and flesh *Lonicera caerulea* berry extracts.

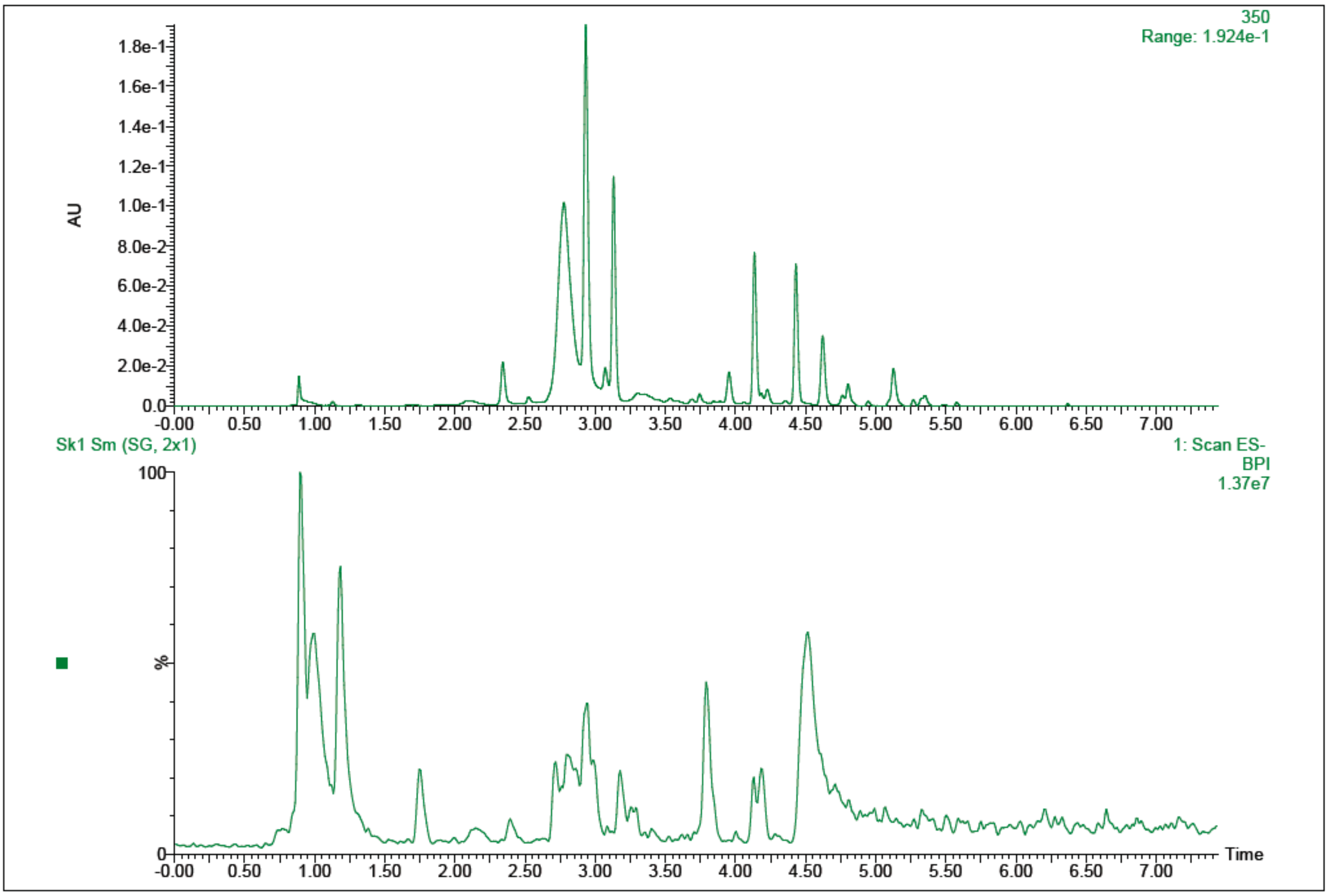

Figure S2. UPLC chromatogram of polyphenolic compounds obtained from the extract of haskap fruit skin (Boreal Beauty).

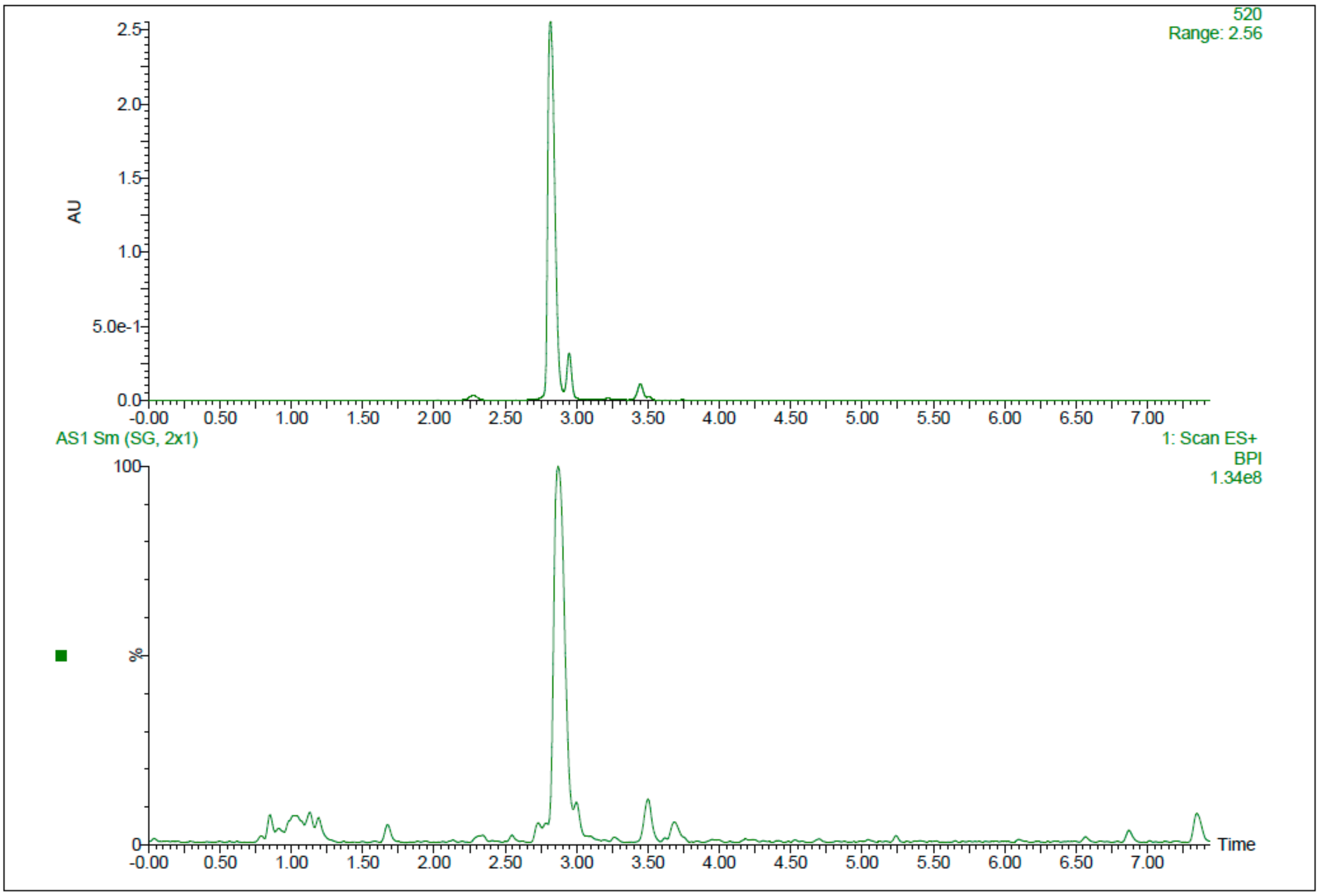

Figure S3. UPLC chromatogram of anthocyanins obtained from the extract of haskap fruit skin (Boreal Beauty).

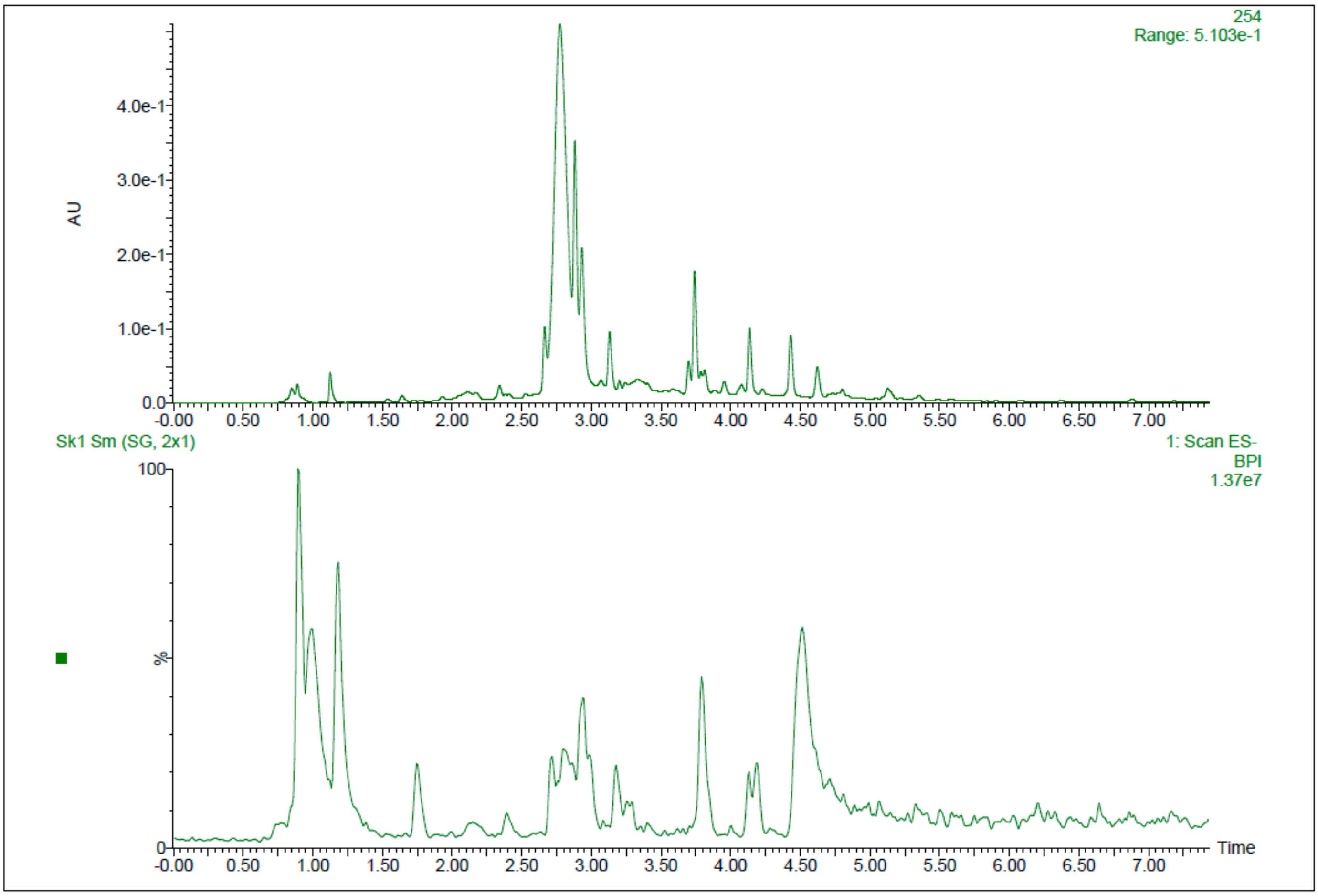

Figure S4. UPLC chromatogram of iridoids obtained from the extract of haskap fruit skin (Boreal Beauty).

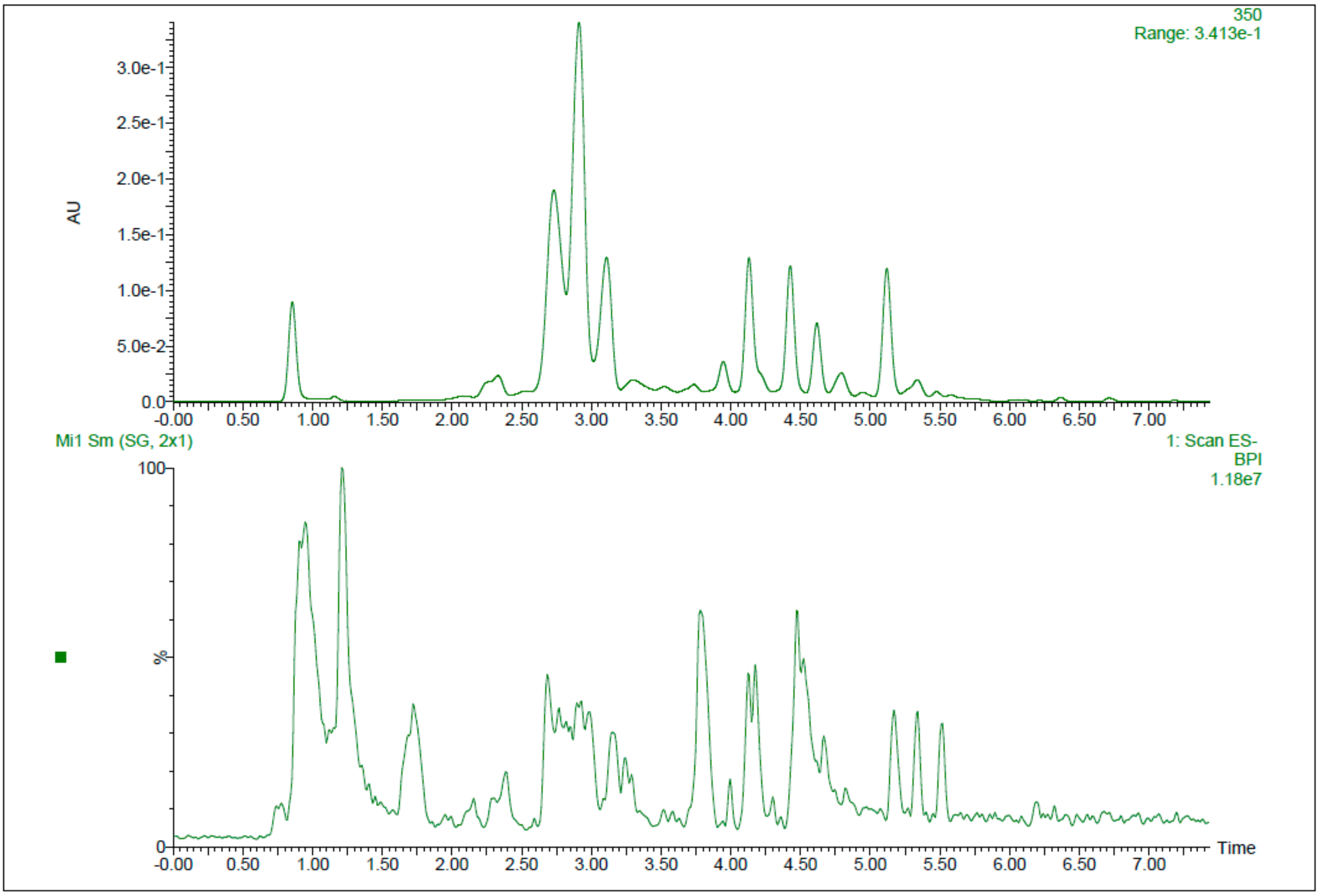

Figure S5. UPLC chromatogram of polyphenolic compounds obtained from the extract of haskap fruit flesh (Boreal Beauty).

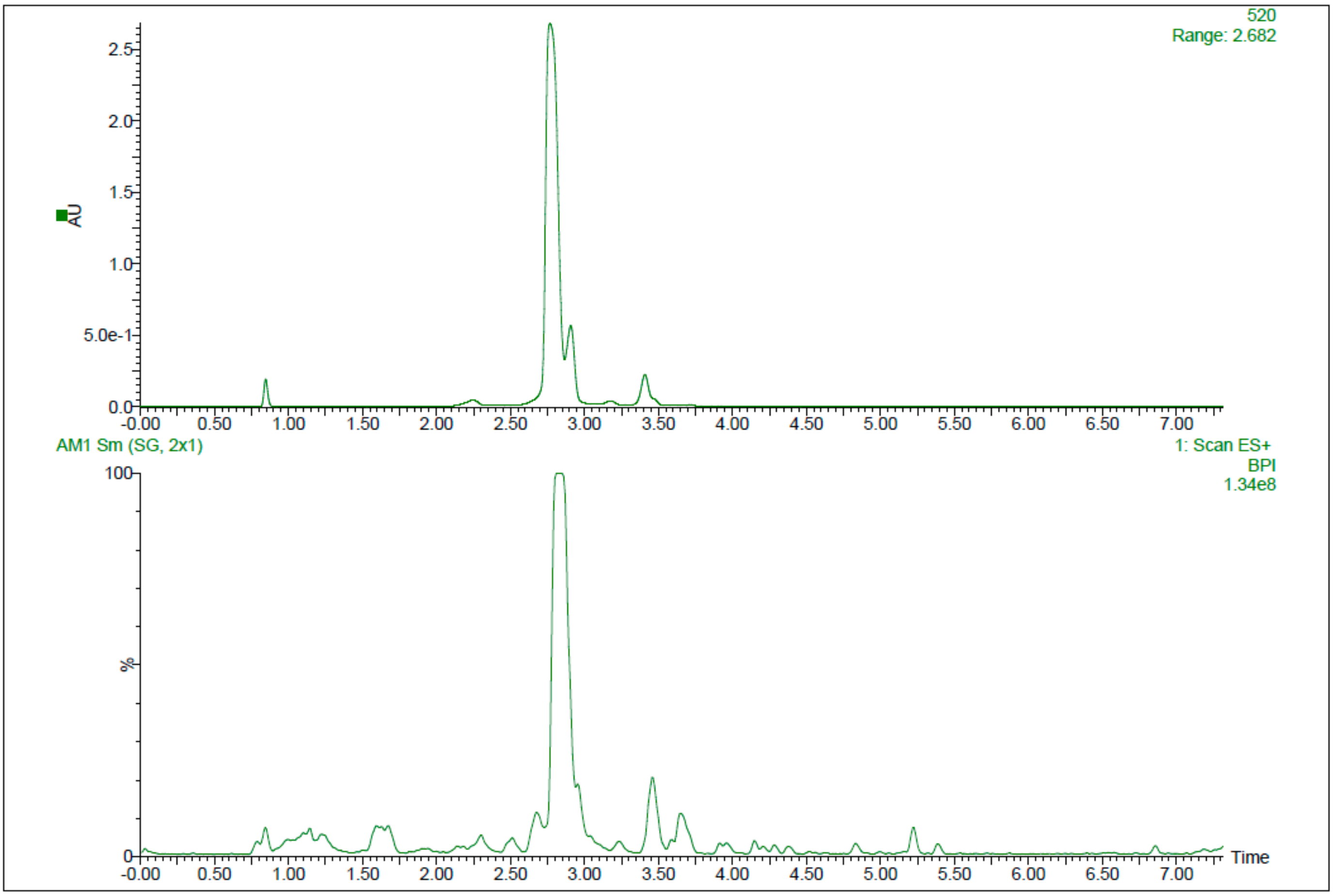

Figure S6. UPLC chromatogram of anthocyanins obtained from the extract of haskap fruit flesh (Boreal Beauty).

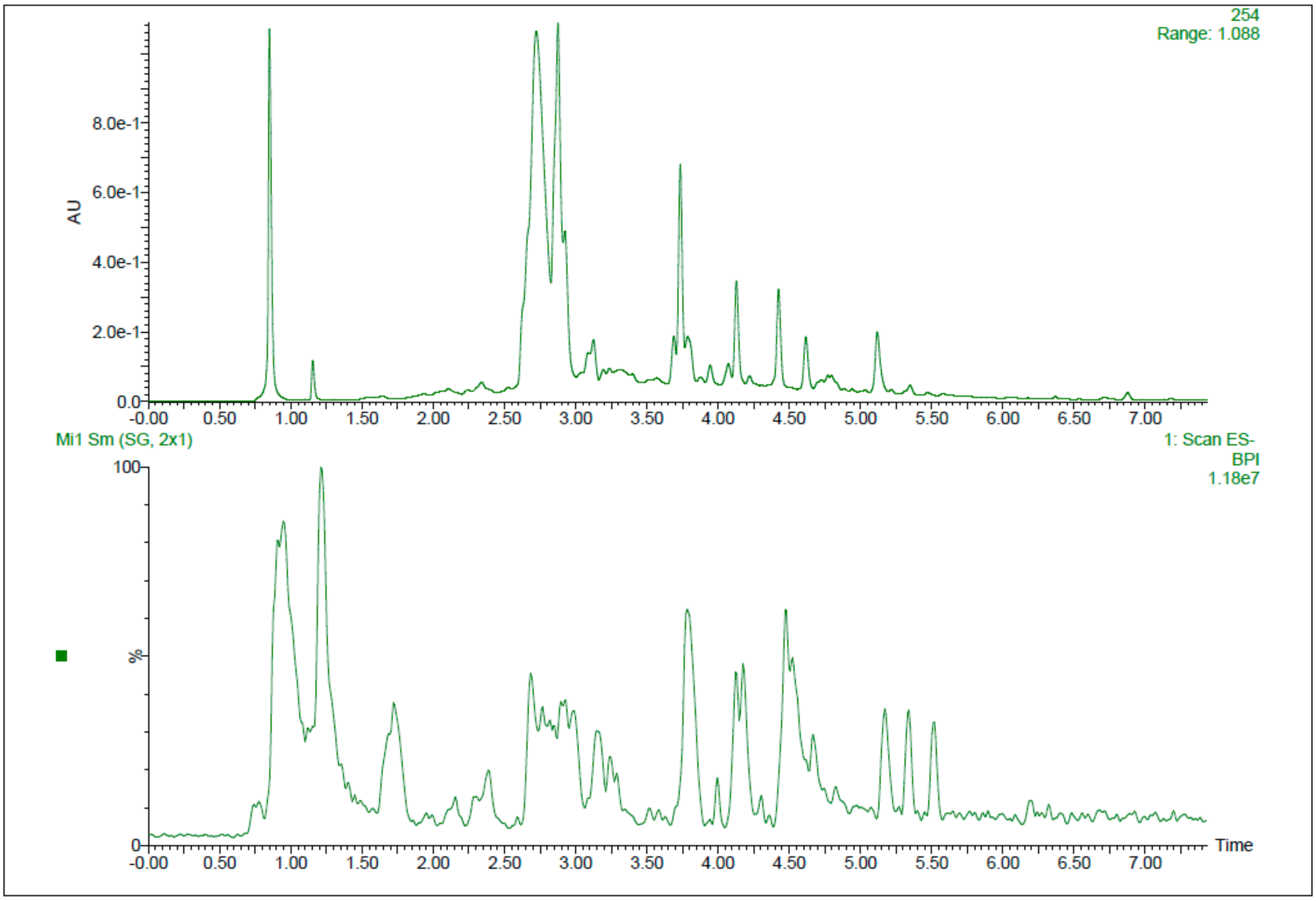

Figure S7. UPLC chromatogram of iridoids obtained from the extract of haskap fruit flesh (Boreal Beauty).
